# Membrane Proteins Have Distinct Fast Internal Motion and Residual Conformational Entropy

**DOI:** 10.1002/anie.202003527

**Published:** 2020-04-30

**Authors:** Evan S. O'Brien, Brian Fuglestad, Henry J. Lessen, Matthew A. Stetz, Danny W. Lin, Bryan S. Marques, Kushol Gupta, Karen G. Fleming, A. Joshua Wand

**Affiliations:** ^1^ Department of Biochemistry & Biophysics Texas A&M University College Station TX 77843 USA; ^2^ Department of Biochemistry & Biophysics University of Pennsylvania Perelman School of Medicine Philadelphia PA 19104 USA; ^3^ Department of Biophysics Johns Hopkins University Baltimore MD 21218 USA; ^4^ Present address: Department of Chemistry Virginia Commonwealth University Richmond VA 23284 USA

**Keywords:** conformational entropy, membrane proteins, NMR spectroscopy, protein folding, side-chain dynamics

## Abstract

The internal motions of integral membrane proteins have largely eluded comprehensive experimental characterization. Here the fast side‐chain dynamics of the α‐helical sensory rhodopsin II and the β‐barrel outer membrane protein W have been investigated in lipid bilayers and detergent micelles by solution NMR relaxation techniques. Despite their differing topologies, both proteins have a similar distribution of methyl‐bearing side‐chain motion that is largely independent of membrane mimetic. The methyl‐bearing side chains of both proteins are, on average, more dynamic in the ps–ns timescale than any soluble protein characterized to date. Accordingly, both proteins retain an extraordinary residual conformational entropy in the folded state, which provides a counterbalance to the absence of the hydrophobic effect. Furthermore, the high conformational entropy could greatly influence the thermodynamics underlying membrane‐protein functions, including ligand binding, allostery, and signaling.

## Graphical abstract

**Two membrane proteins** (one α‐helical and one β‐barrel) have been found, using solution NMR relaxation techniques, to have extraordinary side‐chain motion on the ps–ns timescale in both detergent micelles and lipid bilayers. The extensive side‐chain motion about a highly rigid backbone scaffold is consistent with a high residual conformational entropy and helps explain the stability of the folded state in the absence of the hydrophobic effect.

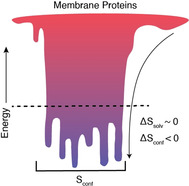

## Introduction

The motions of amino acid side chains of proteins are important for understanding the connection between energetics, structure, and function in these complex macromolecules. For example, the conformational entropy manifested in sub‐nanosecond motion can be a pivotal contribution to the thermodynamics of molecular recognition by proteins.^1^ Furthermore, the dynamical disorder of side chains in the sub‐nanosecond time regime is generally heterogeneously distributed throughout protein molecules, which has significant implications for important aspects of their function.^2^ Three types or classes of motion of methyl‐bearing side chains on this timescale have been discerned thus far by NMR relaxation approaches: highly restricted motion within a single rotameric well (termed the ω‐class), larger excursions within a rotameric well that are accompanied by occasional rotameric interconversion (α‐class); and motions involving more extensive interconversion between two rotameric states (J‐class).^2^, ^3^ These classes of motion are often resolved as distinct statistical distributions of NMR‐derived generalized order parameters.^2^, ^3^ Importantly, ligand binding can result in redistribution of side‐chain motions.^1^, ^4^ A quantitative interpretation of the dynamical response provides insights into the thermodynamics underlying molecular recognition and indicates that the associated changes in conformational entropy can have a significant role in the overall free energy of ligand binding.^1^, ^5^ This view has, thus far, been obtained entirely with water‐soluble proteins.^1^ For a variety of reasons, integral membrane proteins have largely eluded similar investigation. Herein, we report the first comprehensive experimental characterizations of the fast, internal motions of two integral membrane proteins: sensory rhodopsin II (pSRII) and the outer membrane protein W (OmpW). Both proteins display a similar distribution of fast side‐chain motion that is markedly different from that seen in soluble proteins and corresponds to an unusually high residual conformational entropy.

**Figure 1:**
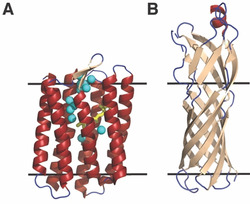
Structural folds of pSRII and OmpW. The crystal structures of pSRII (A, PDB: 1H68)^6^ and OmpW (B, PDB: 2F1T)^7^ are colored by secondary structural elements; α‐helices in red, β‐sheet in tan, and loops in blue. The black lines bracket approximate bilayer‐spanning regions of the proteins. The retinal cofactor of pSRII is drawn as yellow sticks and structural water oxygen atoms are highlighted as cyan spheres.

Our findings are particularly remarkable because OmpW and pSRII represent the extremes of integral membrane protein topologies. pSRII is a 7‐transmembrane α‐helical homolog of G‐protein‐coupled receptor proteins. The structures of isolated pSRII in lipid bilayers have been determined by X‐ray crystallography (Figure 1 a)^6^ and in detergent micelles by solution NMR spectroscopy^8^ and are in good agreement. In contrast, OmpW is a β‐barrel membrane protein found in the outer membrane of *Escherichia coli* and other Gram‐negative bacteria. Like pSRII, the structure of OmpW has been solved using both X‐ray crystallography and solution NMR spectroscopy.^7^, ^9^ It contains 8 transmembrane β‐strands that form a barrel structure in the membrane (Figure 1 b). Half of the inter‐strand connections are short turns while the other four connections are large loop structures. The functions of pSRII and OmpW are also distinct. pSRII mediates negative phototaxis in response to absorption of blue light. The 13‐*trans*‐*cis* isomerization of the attached retinal cofactor activates a two‐component signaling system through its bound transducer partner protein.^10^ Though its precise function has not been confirmed, OmpW has been implicated in multiple cellular processes, such as transport of hydrophobic substrates,^7^ iron uptake,^11^ and antibiotic resistance.^12^ Given these structural and functional differences, the large reservoir of conformational entropy observed here in both proteins points to a general role for fast motions in the thermodynamics governing membrane‐protein stability, folding, and function.

## Results and Discussion

We utilized a recently developed growth and expression strategy^13^ to generate fully ^15^N,^13^CH_3_‐ILVM labeled pSRII in the background of approximately 75–80 % deuteration. The labeling strategy involves expression in H_2_O and avoids the need to “back‐exchange” amide hydrogens that generally plagues the preparation of membrane proteins generated by expression in D_2_O, while maintaining optimal methyl labeling in a background of sufficient deuteration to permit the quantitative measurement of methyl dynamics using cross‐correlated relaxation techniques^14^ (Supporting Information, Figure S1 a,c). The presence of amide ^15^N‐^1^H isotopic labels allows access to local backbone motion and determination of the molecular reorientation time of the protein.^15^ OmpW can be refolded in high yield and thus the traditional approach of expression during growth on bulk D_2_O media permitted a standard labeling strategy for ^15^N,^13^CH_3_‐ILVM labeling in a background of carbon‐bonded deuterium (Supporting Information, Figure S1 b,d). pSRII and OmpW were solubilized for NMR experiments in both detergent micelles (composed of c_7_‐DHPC and SB3‐12, respectively) as well as DMPC lipid bilayers in the form of *q*≈1 bicelles, where *q* is the molar ratio of long‐chain DMPC and short‐chain DHPC. Small‐angle X‐ray scattering (SAXS) was used to confirm that a disc‐like bicelle that maintained bilayer character was preserved in the presence of embedded protein under NMR sample conditions.^16^ It has recently been shown that a feature of the scattering profile can be used to diagnose a transition from a disk‐like, bilayer‐containing bicelle to a mixed bicelle–micelle.^16^ For both pSRII and OmpW, addition of short‐chain lipid results in a right‐shift in a prominent peak feature in the mid‐Q range (approximately 0.15 Å) as the bicelles transition into mixed bicelles–micelles (Supporting Information, Figure S2). The onset of this transition begins below *q*≈0.8 for both proteins. While this trend is clear for pSRII, it is rather muted for OmpW, presumably due to the large extracellular domain that serves to obscure the contribution of the bicelle to the scattering profile. Nevertheless, there is still a clear trend towards larger *Q*_max_ for the main peak feature at lower q ratios (see the inset in Figure S2 b in the Supporting Information). In summary, under the conditions used to obtain NMR relaxation data (*q*=1) both pSRII and OmpW are embedded in true bilayers. Bicelles of *q*=1 are large and therefore tumble more slowly than those traditionally employed in solution NMR studies. While slower tumbling complicates the NMR performance of the embedded protein and makes data acquisition quite challenging, this was judged to be a worthwhile compromise to ensure a true bilayer environment.^17^

We utilized the resonance assignments of pSRII reported by Nietlispach and co‐workers.^8^, ^18^ ^15^N relaxation studies of pSRII in DHPC micelles have indicated that the backbone of pSRII is essentially a rigid scaffold on the sub‐nanosecond timescale.^18^ We repeated ^15^N longitudinal (R_1_) and transverse (R_2_) relaxation experiments to confirm a global reorientation time, which is necessary for the calculation of side‐chain dynamical parameters (21.9±0.9 ns, micelle; 28.9±3.0 ns, bicelle; Supporting Information, Figure S3 a,b). The backbone of pSRII is also largely silent in the slower μs–ms time regime as evidenced by R_1_⋅R_2_ products^19^ and generally flat ^15^N‐dispersion profiles (Supporting Information, Figure S4). Similarly, ^15^N backbone relaxation experiments conducted on OmpW in SB3‐12 detergent micelles allowed the determination of the global reorientation time (24.0±0.9 ns, micelle; 29.9±2.2 ns, bicelle; Supporting Information, Figure S3 c,d) and confirmed the general rigidity of the backbone on both the ns and ms timescales (Supporting Information, Figure S4 e,f).

Methyl cross‐correlated relaxation experiments^14^ were then analyzed and interpreted^20^ in the context of the Lipari–Szabo formalism^21^ yielding the squared generalized order parameter of the methyl‐group symmetry axis (O^2^_axis_) for nearly every resolved methyl resonance in pSRII and OmpW (Supporting Information, Tables S1–S4). O^2^_axis_ values range from one, corresponding to complete rigidity within the molecular frame, to zero, which effectively corresponds to isotropic disorder. Choice of lipid environment can be pivotal for interrogation of membrane‐protein structure, dynamics, and function.^22^ We have incorporated pSRII and OmpW into both detergent micelles as well as lipid bilayers in order to assess the potential role of the membrane environment in membrane‐protein fast‐timescale side‐chain dynamics. Due to their smaller size, micelles result in higher quality and more comprehensive NMR data. Bicelles represent a more native‐like (namely, bilayer) environment, but form larger assemblies with slower macromolecular tumbling that limits the signal‐to‐noise (S/N) of acquired relaxation data. Previous comparisons of membrane‐protein dynamics between lipid conditions have been restricted to limited raw backbone relaxation data.22a, 22b, 22e We aimed here to quantitatively assess lipid environment effects by comparing dynamics directly through determination of methyl order parameters. The NMR chemical shifts of pSRII and OmpW incorporated into micelles and bilayers are very comparable (Supporting Information, Figure S1), and the NOESY patterns^8^ of pSRII prepared in DHPC micelles and DMPC bilayers in the form of bicelles are nearly identical, though maintenance of these parameters are not necessarily indicative of conserved dynamics.

To test this, we compared methyl O^2^_axis_ values for both proteins in the two environments. An excellent correlation of O^2^_axis_ values between the two lipid environments was observed for those methyl probes having data of suitable quality and could be mapped between both membrane mimetics (R^2^=0.81 and 0.96 for pSRII and OmpW, respectively; Supporting Information, Figure S5).

These comparisons suggest that fast‐timescale side‐chain motions are less sensitive to the membrane mimetic than previously anticipated.22a, 22e Due to the apparent lack of dependence of fast‐timescale dynamics on lipid environment for both membrane‐protein systems, we pursued more detailed investigations of higher quality data obtainable from micelle‐incorporated proteins. 90 of 146 ILVM methyl probes in pSRII and 55 of 65 ILVM methyl probes in OmpW were adequately resolved and of sufficient S/N to give O^2^_axis_ values with an average precision of ±0.038 for pSRII and ±0.019 for OmpW (Supporting Information, Figure S6).

**Figure 2:**
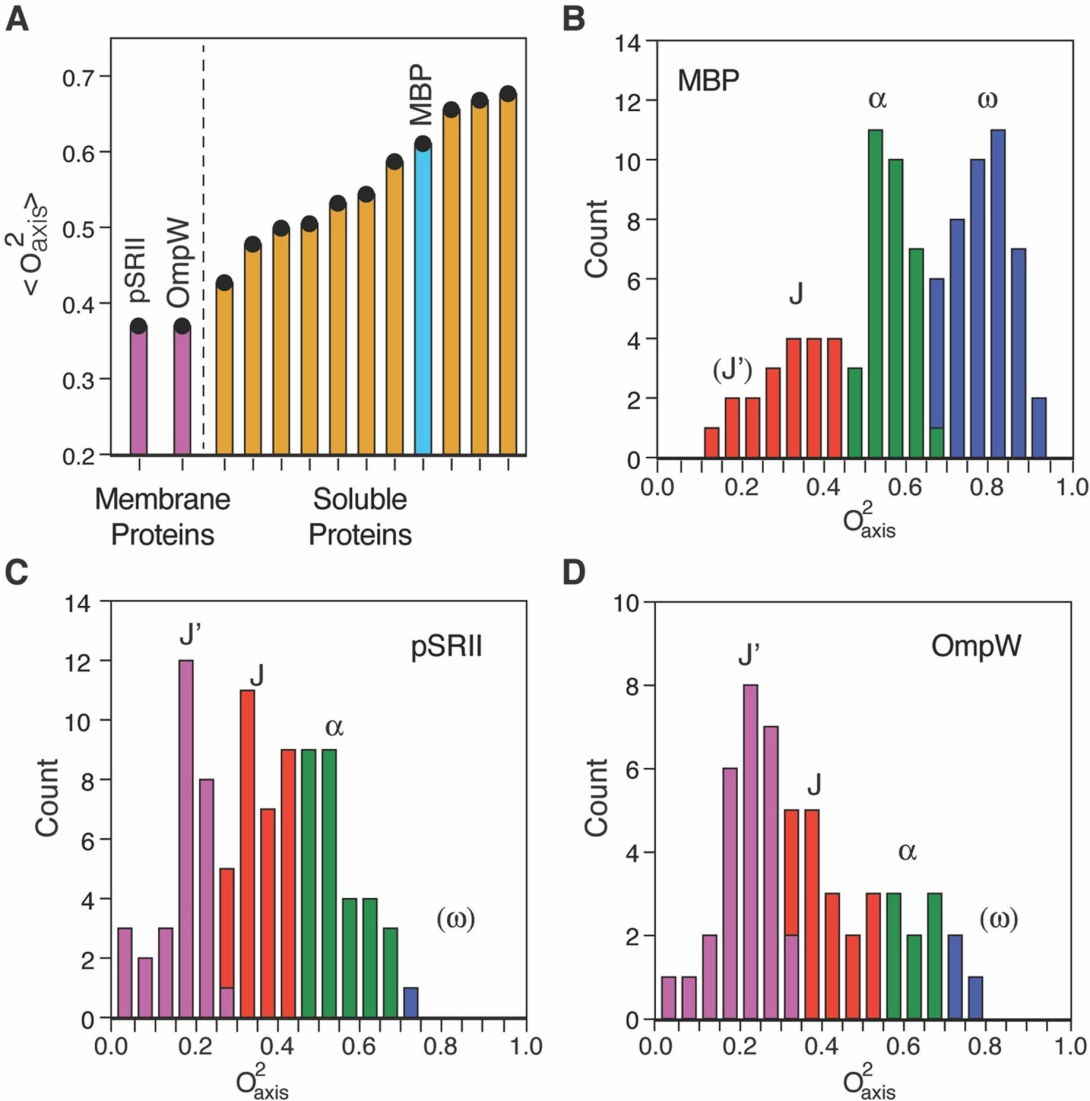
The distribution of fast side‐chain motion in membrane proteins is distinct from their soluble counterparts. A) Average methyl‐symmetry axis order parameters seen in soluble proteins and integral membrane proteins. Purple bars correspond to pSRII (50 °C) and OmpW (40 °C). The cyan bar corresponds to maltose binding protein (MBP), chosen to illustrate a representative distribution of soluble protein methyl‐dynamics in (B); Orange bars correspond to other proteins spanning the known range of soluble protein methyl‐bearing side‐chain dynamics. The source data is described in Table S6 in the Supporting Information. B) Distribution of O^2^_axis_ values in MBP (Supporting Information, Table S5). Shown to illustrate the distributions commonly seen in soluble proteins, particularly the segregation into three classes of underlying motion (J, red; α, green; ω, blue) as well as the emergence of a new class of motion in the two integral membrane proteins (J′, purple) seen in pSRII (C) and OmpW (D). Class boundaries were calculated using the k‐means clustering algorithm. The rigid ω‐class is effectively absent in both pSRII and OmpW. Class centers are indicated by the position of the class label. The newly observed J′‐class is centered on an O^2^_axis_ of approximately 0.21. Only O^2^_axis_ values with error≤±0.1 are included.

Both pSRII and OmpW display internal protein motion that is distinct from that previously observed for soluble proteins. The average O^2^_axis_ of pSRII is 0.36, which is unusually low and has not been observed previously in structured proteins (Figure 2 a). Ca^2+^‐saturated calmodulin (CaM) is the next most dynamic protein characterized in this way (⟨O^2^_axis_⟩≈0.43), largely due to depopulation of the aforementioned ω‐band motional class.^2^, ^3^, 4b The cross‐correlated relaxation experiments were best carried out at 50 °C for pSRII, which is somewhat higher than corresponding studies with the other proteins shown in Figure 2 a. Experiments at a more comparable but less optimal temperature of 35 °C gave an average change in O^2^_axis_ of +0.055, shifting the ⟨O^2^_axis_⟩ at the lower temperature to 0.415. The temperature dependence (dO^2^_axis_/dT) of pSRII is −0.0037 K^−1^, which is slightly larger than that observed for a calmodulin complex^23^ and ubiquitin^24^ and possibly hints at subtle differences in effective heat capacity between soluble and integral membrane proteins. Surprisingly, a comparable analysis of the topologically distinct OmpW demonstrates that it is also extraordinarily dynamic, with an average side‐chain methyl O^2^_axis_ value of 0.36 (Figure 2 a). Both membrane proteins measured in this manner are far more dynamic on average than any wild‐type soluble protein investigated to date (Figure 2 a).

OmpW and pSRII also have a striking distribution of sub‐nanosecond methyl‐bearing side‐chain motion (Figure 2 c,d) that is distinct from that typically observed in soluble proteins (Figure 2 b). There is almost a complete absence of rigid methyl‐bearing side chains of the so‐called ω‐class of motion, which is generally significantly populated in soluble proteins (Figure 2 b). The populations of the J‐ and α‐classes are roughly equivalent in OmpW and pSRII. Furthermore, a previously undocumented and qualitatively distinct class of motion is observed in these two membrane proteins. Approximately one‐third of the methyl‐bearing side chains exhibit an unusually high degree of dynamic disorder on the sub‐nanosecond timescale. Analysis of the distribution of O^2^_axis_ values using a Bayesian statistical approach^3^ suggests two and three classes of motion with nearly the same likelihood. The three‐class model seems more appropriate as two of the classes roughly match the centers of the J‐ and α‐classes seen in soluble proteins; the centers of the J‐ and α‐classes were further refined with a k‐means clustering methodology and found to be 0.36 and 0.55, respectively, for pSRII. The J‐ and α‐class centers for OmpW are 0.42 and 0.66, respectively. This roughly matches results for soluble proteins.^2^, ^3^ Regardless, a new motional class, which we term J′, is centered at an O^2^_axis_ value of approximately 0.21 for both proteins. Such a low order parameter requires that two or more rotamers of each torsion angle are extensively sampled. Though molecular dynamics simulations do not reproduce the experimental O^2^_axis_ values quantitatively (Supporting Information, Figure S7), the highly dynamic J′ band of motions is well represented in the simulations of both proteins in the bilayer. An analysis of those residues comprising the J′‐class in pSRII that are reasonably reproduced by simulation reinforces the notion that extensive rotameric interconversion of multiple torsion angles is required to achieve such low order parameters. This is in accord with theoretical considerations.^25^ The J′‐class is also enriched relative to the overall average in methionine, leucine, and isoleucine residues (73 % versus 55 %), which have two or more torsion angles to sample, and diminished in valine (27 % versus 39 %; only one non‐terminal valine residue (Val101) is in the J′‐class).

**Figure 3:**
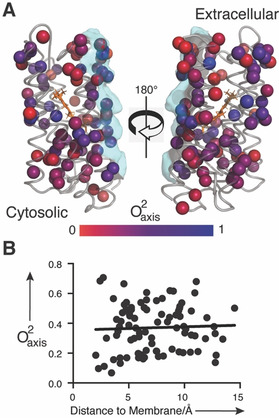
Spatial distribution of fast side‐chain motion in pSRII. A) A ribbon representation of the crystal structure of pSRII (PDB: 1H68)^6^ on which spheres representing methyl probes are colored according to O^2^_axis_ value using a gradient of red for most dynamic (O^2^_axis_=0) to blue for most rigid (O^2^_axis_=1). The cytosolic and extracellular faces of the protein are noted. The retinal cofactor is shown as orange sticks and the surface of pSRII that forms the interface with its hTRII binding partner is shown as semi‐transparent cyan. B) The lack of apparent correlation of methyl dynamics with the distance to nearest lipid atom (R^2^=0.002) further shows that methyl dynamics are heterogeneously distributed in the protein structure.

We took advantage of previous methyl‐group assignments in pSRII^18^ to investigate the structural context of its side‐chain dynamics. Methyl probes are depicted as spheres on the crystal structure^6^ and colored in a O^2^_axis_ gradient ranging from zero (red) to one (blue, Figure 3 a). As in soluble‐protein systems^2^ there is a heterogeneous distribution of disorder in O^2^_axis_ with no statistically significant (*p*‐value>0.2) spatial clustering of motional classes as revealed by the k‐means approach.^26^ One might predict that methyl‐bearing side chains exposed to lipid (or detergent) will tend to be more dynamic than residues more deeply buried in the structural core of the protein. However, pSRII does not display such “surface molten” behavior but rather has both highly dynamic and comparatively rigid probes exposed at the membrane (Figure 3 b) and aqueous surfaces of the protein (Supporting Information, Figure S8 a). Methyl probes near what will serve as the interface with the transducer binding partner^27^ do not generally occupy the highly dynamic J′ motional class (⟨O^2^_axis_⟩=0.45), though several relatively dynamic methyl probes do sit at the extracellular side of the interface. The region surrounding the retinal cofactor is not well sampled by well‐determined methyl probes; many are sufficiently broadened by dipolar interactions with the ^1^H‐rich retinal cofactor to degrade the quality of the relaxation measurements. However, Met109 C*ϵ*, which is approximately 3.5 Å away and lies roughly perpendicular to the plane of conjugated double bonds, is one of the most rigid methyl groups in the protein (O^2^_axis_=0.68). The unavailability of deuterated SB3‐12 detergent thwarted collection of methyl assignment of sufficient quality to access resonance assignments and thus prevented a similar structural analysis of OmpW.

**Figure 4:**
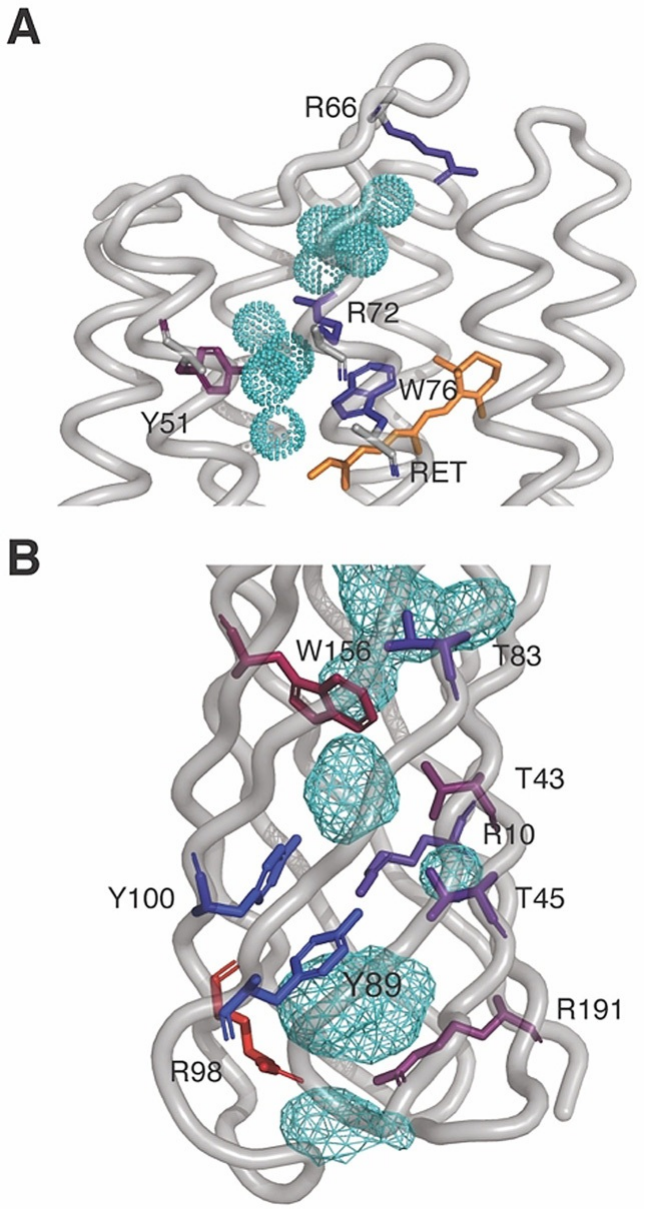
Polar cores of pSRII and OmpW. A) Structural water oxygen atoms in the crystal structure (PDB: 1H68)^6^ of pSRII are shown as cyan dotted spheres. Polar residues with side chains within hydrogen‐bonding distance of structural water molecules are depicted as sticks and are colored according to O^2^ values extracted from molecular dynamics simulations according to the same red (O^2^=0.0) to blue (O^2^=1.0) scaling used in Figure 3. Order parameters were calculated using the Nϵ‐Hϵ bond vector for arginine, the Cβ‐Cγ2 vector for threonine, and the Cδ1‐Hδ1 vector for tyrosine and tryptophan. The average polar side chain O^2^ in the core of pSRII is 0.70. B) The interior cavities of OmpW (PDB: 2MHL)^9^ are colored as cyan mesh. The average polar side chain O^2^ in the core of OmpW is 0.57.

The absence the ω‐class of motions and emergence of the new highly dynamic J′‐class indicates that the side chains are essentially fluid and raises a question of what interactions drive the formation of a stable tertiary structure. In this regard, it is interesting to note that small internal clusters of water molecules are commonly observed in crystal structures of GPCRs, which are structurally homologous to pSRII, and have been argued to play an important structural role as well as being actively involved in transitions between active/inactive states.^28^ In pSRII, crystallographic waters are clustered between the extracellular β‐sheet and the retinal and appear to electrostatically support a buried arginine (Arg72, Figure 4 A). This large water network lies near the extracellular face and is divided by a pair of hydrophobic residues (Ile197 and Val194) and Arg72, which is involved in hydrogen‐bonding interactions with both water clusters. Experimental insight provided by methyl dynamics near this structural water network is limited to Val194 (O^2^_axis_=0.88±0.11; not included in detailed statistics or Figure 2 due to relatively high uncertainty) and Ile197 (O^2^_axis_=0.14). The presence of the water network in solution was confirmed using a methyl ^13^C‐NOESY experiment. Strong negative NOE cross‐peaks between the water resonance and methyl groups surrounding both deeply buried water clusters are seen (Supporting Information, Figure S9). The negative sign and strength of the NOEs indicate a relatively rigid protein–water interaction. Met15, which is proximal to the most buried water in cluster 2, Ile197, which lies between the water clusters, and Val68, which is near the extracellular‐facing cluster 1, all display negative NOEs to the water resonance (Supporting Information, Figure S9). While many other methyl probes display NOEs to the water resonance, most are solvent exposed or potentially contaminated by a nearby hydroxy‐group‐containing residue, which can potentially relay magnetization to water by hydrogen exchange.^29^ Unfortunately, the pSRII‐containing micelle particle tumbles too slowly to permit measurement of the counterpart ROE that could potentially allow a more precise evaluation of the residence time of these buried waters.^30^ Notwithstanding the limitations of molecular dynamics to quantitatively reproduce experimentally determined order parameters in the current context, it is important to note that simulation suggests that nearly all buried polar side chains interacting with buried waters in both pSRII and OmpW are quite rigid on the fast‐timescale, especially when compared to the dynamics of methyl‐bearing side chains in each protein (Figure 4).

## Conclusion

**Figure 5:**
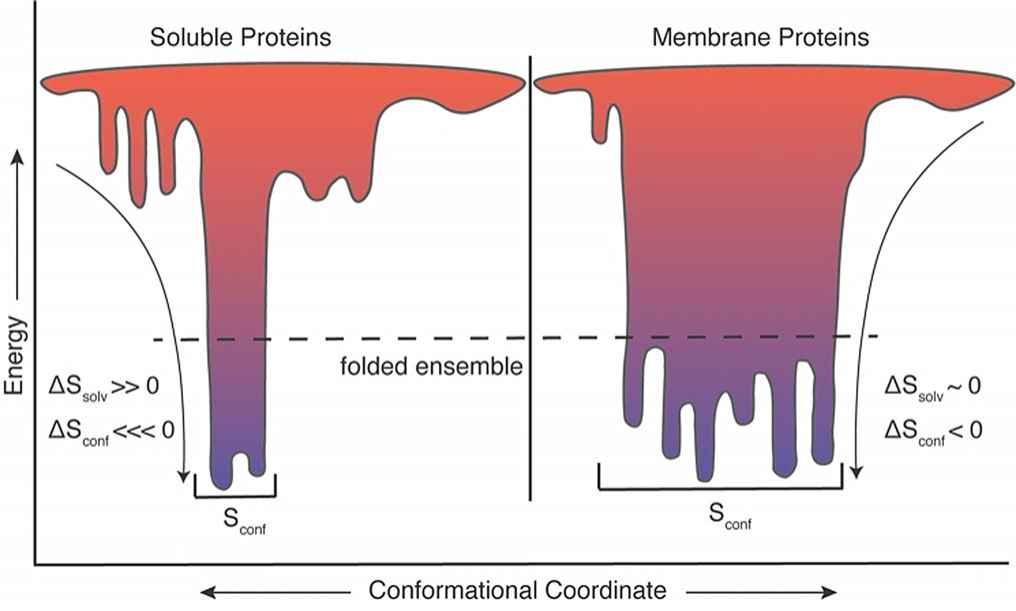
Supported hypothesis that soluble and membrane proteins utilize solvent and conformational entropies differently to stabilize their folded state ensembles. The folded state of soluble proteins is favored by a large increase in the entropy of water (Δ*S*_solv_, change in solvent entropy) and disfavored by a loss of chain and main‐ and side‐chain entropy (Δ*S*_conf_, change in conformational entropy.^32^, ^36^ In contrast, integral membrane proteins appear to mitigate the absence of a favorable change in solvent water entropy through a diminished unfavorable contribution of reduced side‐chain conformational entropy.

In summary, the first comprehensive studies of fast side‐chain motion in integral membrane proteins presented here have revealed the existence of extensive sampling of microstates on the sub‐nanosecond timescale and point to a relatively high residual conformational entropy in pSRII and OmpW. pSRII and OmpW represent the topological extremes of integral membrane proteins and yet have the same dynamical signature. As such, they begin to suggest that their unusual dynamical character may be a general feature of integral membrane proteins. Future work will address this question. Nevertheless, the high residual conformational entropy present in the native folded structures of these two proteins potentially impacts their stability, folding, and function. For example, the stability of the folded state of an integral membrane protein,^31^ as for all proteins,^32^ results from a balance of forces. For soluble proteins, a dominant contribution to the stability of the folded state is the so‐called hydrophobic effect that arises from the release of associated water from surfaces that are buried upon folding.^32^ With respect to integral membrane proteins, this stabilizing entropic contribution from water is absent once the polypeptide chain has been inserted into the membrane. The water‐to‐bilayer transfer of the unfolded polypeptide chain from water into the lipid bilayer is subsequently followed by folding to the final native state.^31^, ^33^ Thus, in contrast to soluble proteins, folding of the polypeptide chain within the membrane lacks the general driving force of the hydrophobic effect, that is, the gain in solvent water entropy as the protein adopts a compact structure with less accessible surface area. The results presented here suggest that the extraordinary residual conformational entropy of the folded state of membrane proteins helps avoid some of the penalty of organizing the tertiary fold within the membrane (Figure 5). The large folding free energy of OmpW in large unilamellar vesicles (approximately 18 kcal mol^−1^)^34^ is also consistent with a minimal side‐chain entropy penalty imposed by adoption of the folded state. Though the Δ*G* for folding has not been determined for pSRII directly as for OmpW, chemical denaturation studies indicate that it is also quite stable^35^ and NMR spectra collected at elevated temperature reinforce that view.

The unusual dynamical character of pSRII is also relevant to our understanding of its mechanism of action and may serve as an informative counterpoint to GPCRs that are at the center of multicellular signaling. pSRII is a simple discrete binary switch and is apparently made so, in part, by a significant residual conformational entropy in the inactivated state that deepens the free energy well in which it resides. Conformational entropy manifested in methyl‐group motions, in concert with the water‐mediated polar core interactions, simplifies its average structural character and would make a discrete transition upon activation possible. In contrast, non‐olfactory GPCRs do not appear to occupy a localized structural ensemble and the lack of a single biophysical species has made detailed dynamical studies difficult.^37^ ^13^C‐methionine methyl labeling indicates that unliganded β_2_ adrenergic receptor (β_2_AR) displays extensive conformational exchange on the NMR chemical shift timescale (namely, millisecond) indicating that barriers between distinct functional states are large.^38^ GPCRs that are required to respond to an array of signaling events (namely, binding of agonists, antagonists, G‐proteins, G‐protein coupled receptor kinases, and β‐arrestins) cycle between their various functional states. Indeed, a recent examination of A_2A_R reinforces this conclusion.^39^ Fast‐timescale changes in structural water molecule positions near the retinal binding site in bacteriorhodopsin have been shown to facilitate these discrete transitions in functional state;^40^ highly dynamic methyl groups may be playing a similar role. Finally, perturbations in these novel patterns of methyl dynamics in response to variations in membrane environment, binding partners, ligands, amongst others, are potentially functionally and thermodynamically important, and should yield further insights into the role of fast dynamics in membrane‐protein function and interaction.

## Conflict of interest

The authors declare no conflict of interest.

## Supporting information

As a service to our authors and readers, this journal provides supporting information supplied by the authors. Such materials are peer reviewed and may be re‐organized for online delivery, but are not copy‐edited or typeset. Technical support issues arising from supporting information (other than missing files) should be addressed to the authors.

Supplementary

## References

[1] J. A. Caro , K. W. Harpole , V. Kasinath , J. Lim , J. Granja , K. G. Valentine , K. A. Sharp , A. J. Wand , Proc. Natl. Acad. Sci. USA 2017, 114, 6563–6568. 28584100 10.1073/pnas.1621154114 PMC5488930

[2] T. I. Igumenova , K. K. Frederick , A. J. Wand , Chem. Rev. 2006, 106, 1672–1699. 16683749 10.1021/cr040422h PMC2547146

[3] K. A. Sharp , V. Kasinath , A. J. Wand , Proteins Struct. Funct. Bioinf. 2014, 82, 2106–2117. 10.1002/prot.24566 PMC4142109 24677353

[4a] A. L. Lee , S. A. Kinnear , A. J. Wand , Nat. Struct. Biol. 2000, 7, 72–77; 10625431 10.1038/71280

[4b] K. K. Frederick , M. S. Marlow , K. G. Valentine , A. J. Wand , Nature 2007, 448, 325–329. 17637663 10.1038/nature05959 PMC4156320

[5a] A. J. Wand , K. A. Sharp , Annu. Rev. Biophys. 2018, 47, 41–61; 29345988 10.1146/annurev-biophys-060414-034042 PMC7071556

[5b] V. Kasinath , K. A. Sharp , A. J. Wand , J. Am. Chem. Soc. 2013, 135, 15092–15100. 24007504 10.1021/ja405200u PMC3821934

[6] A. Royant , P. Nollert , K. Edman , R. Neutze , E. M. Landau , E. Pebay-Peyroula , J. Navarro , Proc. Natl. Acad. Sci. USA 2001, 98, 10131–10136. 11504917 10.1073/pnas.181203898 PMC56927

[7] H. Hong , D. R. Patel , L. K. Tamm , B. van den Berg , J. Biol. Chem. 2006, 281, 7568–7577. 16414958 10.1074/jbc.M512365200

[8] A. Gautier , H. R. Mott , M. J. Bostock , J. P. Kirkpatrick , D. Nietlispach , Nat. Struct. Mol. Biol. 2010, 17, 768–774. 20512150 10.1038/nsmb.1807 PMC2923064

[9] R. Horst , P. Stanczak , K. Wuthrich , Structure 2014, 22, 1204–1209. 25017731 10.1016/j.str.2014.05.016 PMC4150354

[10] O. S. Mironova , R. G. Efremov , B. Person , J. Heberle , I. L. Budyak , G. Buldt , R. Schlesinger , FEBS Lett. 2005, 579, 3147–3151. 15919078 10.1016/j.febslet.2005.05.010

[11] M. Catel-Ferreira , S. Marti , L. Guillon , L. Jara , G. Coadou , V. Molle , E. Bouffartigues , G. Bou , I. Shalk , T. Jouenne , X. Vila-Farres , E. De , FEBS Lett. 2016, 590, 224–231. 26823169 10.1002/1873-3468.12050

[12] X. M. Lin , J. N. Yang , X. X. Peng , H. Li , J. Proteome Res. 2010, 9, 5952–5959. 20718490 10.1021/pr100740w

[13] E. S. O'Brien , D. W. Lin , B. Fuglestad , M. A. Stetz , T. Gosse , C. Tommos , A. J. Wand , J. Biomol. NMR 2018, 71, 263–273. 30073492 10.1007/s10858-018-0200-7 PMC6165672

[14] H. Sun , L. E. Kay , V. Tugarinov , J. Phys. Chem. B 2011, 115, 14878–14884. 22040035 10.1021/jp209049k

[15] V. A. Jarymowycz , M. J. Stone , Chem. Rev. 2006, 106, 1624–1671. 16683748 10.1021/cr040421p

[16] T. A. Caldwell , S. Baoukina , A. T. Brock , R. C. Oliver , K. T. Root , J. K. Krueger , K. J. Glover , D. P. Tieleman , L. Columbus , J. Phys. Chem. Lett. 2018, 9, 4469–4473. 30024762 10.1021/acs.jpclett.8b02079 PMC6353637

[17] A. Piai , Q. S. Fu , J. Dev , J. J. Chou , Chem. Eur. J. 2017, 23, 1361–1367. 27747952 10.1002/chem.201604206 PMC5272838

[18] A. Gautier , J. P. Kirkpatrick , D. Nietlispach , Angew. Chem. Int. Ed. 2008, 47, 7297–7300; 10.1002/anie.200802783 18677733

[19] J. M. Kneller , M. Lu , C. Bracken , J. Am. Chem. Soc. 2002, 124, 1852–1853. 11866588 10.1021/ja017461k

[20] M. A. Stetz , J. A. Caro , S. Kotaru , X. J. Yao , B. S. Marques , K. G. Valentine , A. J. Wand in Biological Nmr, Pt B, Vol. 615 (Ed.: A. J. Wand), 2019, pp. 237–284. 10.1016/bs.mie.2018.09.010 PMC6364297 30638531

[21] G. Lipari , A. Szabo , J. Am. Chem. Soc. 1982, 104, 4546–4559.

[22a] F. Hagn , M. Etzkorn , T. Raschle , G. Wagner , J. Am. Chem. Soc. 2013, 135, 1919–1925; 23294159 10.1021/ja310901f PMC3566289

[22b] Y. L. Song , K. F. Mittendorf , Z. W. Lu , C. R. Sanders , J. Am. Chem. Soc. 2014, 136, 4093–4096; 24564538 10.1021/ja4114374 PMC3985881

[22c] L. Frey , N. A. Lakomek , R. Riek , S. Bibow , Angew. Chem. Int. Ed. 2017, 56, 380–383; 10.1002/anie.201608246 PMC6680326 27882643

[22d] D. P. Staus , L. M. Wingler , D. Pichugin , R. S. Prosser , R. J. Lefkowitz , J. Biol. Chem. 2019, 294, 13218–13223; 31362983 10.1074/jbc.AC119.009848 PMC6737212

[22e] S. Bibow , S. Hiller , FEBS J. 2019, 286, 1610–1623. 30133960 10.1111/febs.14639

[23] A. L. Lee , A. J. Wand , Nature 2001, 411, 501–504. 11373686 10.1038/35078119

[24] X. J. Song , P. F. Flynn , K. A. Sharp , A. J. Wand , Biophys. J. 2007, 92, L43–L45. 17218465 10.1529/biophysj.106.102160 PMC1861776

[25a] G. Lipari , A. Szabo , J. Am. Chem. Soc. 1982, 104, 4559–4570;

[25b] R. J. Wittebort , A. Szabo , J. Chem. Phys. 1978, 69, 1722–1736.

[26] Y. Fu , V. Kasinath , V. R. Moorman , N. V. Nucci , V. J. Hilser , A. J. Wand , J. Am. Chem. Soc. 2012, 134, 8543–8550. 22452540 10.1021/ja3004655 PMC3415598

[27a] A. Ishchenko , E. Round , V. Borshchevskiy , S. Grudinin , I. Gushchin , J. P. Klare , A. Remeeva , V. Polovinkin , P. Utrobin , T. Balandin , M. Engelhard , G. Buldt , V. Gordeliy , Sci. Rep. 2017, 7, 41811; 28165484 10.1038/srep41811 PMC5292967

[27b] S. Hippler-Mreyen , J. P. Klare , A. A. Wegener , R. Seidel , C. Herrmann , G. Schmies , G. Nagel , E. Bamberg , M. Engelhard , J. Mol. Biol. 2003, 330, 1203–1213. 12860139 10.1016/s0022-2836(03)00656-9

[28] S. G. Yuan , S. Filipek , K. Palczewski , H. Vogel , Nat. Commun. 2014, 5, 4733. 25203160 10.1038/ncomms5733

[29a] C. Jorge , B. S. Marques , K. G. Valentine , A. J. Wand , Methods Enzymol. 2019, 615, 77–101; 30638541 10.1016/bs.mie.2018.09.040 PMC6358200

[29b] G. Otting , Prog. Nucl. Magn. Reson. Spectrosc. 1997, 31, 259–285. 10.1016/j.pnmrs.2016.11.001 28283085

[30] G. Otting , E. Liepinsh , K. Wuthrich , Science 1991, 254, 974–980. 1948083 10.1126/science.1948083

[31] S. H. White , W. C. Wimley , Annu. Rev. Biophys. Biomol. Struct. 1999, 28, 319–365. 10410805 10.1146/annurev.biophys.28.1.319

[32] K. A. Dill , Biochemistry 1990, 29, 7133–7155. 2207096 10.1021/bi00483a001

[33] K. G. Fleming , Annu. Rev. Biophys. 2014, 43, 233–255. 24895854 10.1146/annurev-biophys-051013-022926

[34] C. P. Moon , N. R. Zaccai , P. J. Fleming , D. Gessmann , K. G. Fleming , Proc. Natl. Acad. Sci. USA 2013, 110, 4285–4290. 23440211 10.1073/pnas.1212527110 PMC3600475

[35] Y. L. Tan , J. Mitchell , J. Klein-Seetharaman , D. Nietlispach , J. Mol. Biol. 2018, 430, 4068–4086. 30098339 10.1016/j.jmb.2018.07.031

[36] H. Frauenfelder , S. G. Sligar , P. G. Wolynes , Science 1991, 254, 1598–1603. 1749933 10.1126/science.1749933

[37a] C. Tian , R. M. Breyer , H. J. Kim , M. D. Karra , D. B. Friedman , A. Karpay , C. R. Sanders , J. Am. Chem. Soc. 2005, 127, 8010–8011; 15926814 10.1021/ja051161b

[37b] S. H. Park , F. Casagrande , B. B. Das , L. Albrecht , M. Chu , S. J. Opella , Biochemistry 2011, 50, 2371–2380. 21323370 10.1021/bi101568j PMC3236025

[38] R. Nygaard , Y. Zou , R. O. Dror , T. J. Mildorf , D. H. Arlow , A. Manglik , A. C. Pan , C. W. Liu , J. J. Fung , M. P. Bokoch , F. S. Thian , T. S. Kobilka , D. E. Shaw , L. Mueller , R. S. Prosser , B. K. Kobilka , Cell 2013, 152, 532–542. 23374348 10.1016/j.cell.2013.01.008 PMC3586676

[39] L. D. Clark , I. Dikiy , K. Chapman , K. E. J. Rodstrom , J. Aramini , M. V. LeVine , G. Khelashvili , S. G. F. Rasmussen , K. H. Gardner , D. M. Rosenbaum , eLife 2017, 6, 28505. 10.7554/eLife.28505 PMC5650471 28984574

[40] P. Nogly , T. Weinert , D. James , S. Carbajo , D. Ozerov , A. Furrer , D. Gashi , V. Borin , P. Skopintsev , K. Jaeger , K. Nass , P. Bath , R. Bosman , J. Koglin , M. Seaberg , T. Lane , D. Kekilli , S. Brunle , T. Tanaka , W. T. Wu , C. Milne , T. White , A. Barty , U. Weierstall , V. Panneels , E. Nango , S. Iwata , M. Hunter , I. Schapiro , G. Schertler , R. Neutze , J. Standfuss , Science 2018, 361, eaat0094. 29903883 10.1126/science.aat0094

