# Supplementary material for: Membrane Proteins Have Distinct Fast Internal Motion and Residual Conformational Entropy
Source: Angew Chem Int Ed Engl. 2020 Apr 30;59(27):11108–14. doi: 10.1002/anie.202003527 (PMC7318686; doi:10.1002/anie.202003527)
Supplement: Supplementary file 1 — Supplementary [file ANIE-59-11108-s001.pdf]

Angewandte Chemie
Eine Zeitschrift der Gesellschaft Deutscher Chemiker

Supporting Information

Membrane Proteins Have Distinct Fast Internal Motion and Residual Conformational Entropy

*Evan S. O'Brien, Brian Fuglestad, Henry J. Lessen, Matthew A. Stetz, Danny W. Lin, Bryan S. Marques, Kushol Gupta, Karen G. Fleming, and A. Joshua Wand**

anie_202003527_sm_miscellaneous_information.pdf

Materials and Methods

Sample Preparation

Expression of labeled pSRII – A pET11a vector containing the gene for sensory rhodopsin II from *Natronomonas pharaonis* with a C-terminal histidine tag was freshly transformed into BL21(DE3) competent *E. coli* cells. An overnight culture was used to inoculate 1 L of 1.5x M9 medium in H_2O with ^{15}N labeling at an initial OD_{600} of ~0.1 at 37°C, pH 7.4, and 40% dissolved oxygen concentration. This first phase of growth utilized per liter: 1 g $^{15}NH_4Cl$, 2 g glucose, 200 mg yeast extract, 0.24 g $MgSO_4$, 15 mg $CaCl_2$, 15 mg $ZnCl_2$, 15 mg $FeSO_4$ and appropriate antibiotic. Temperature, pH, and oxygen were maintained or monitored in a New Brunswick BioFlo 115 cell culture system within a 3 L vessel. Cells were grown to saturation (OD_{600} ~ 2.4) as judged by the spike in dissolved oxygen concentration, at which point the temperature was dropped to 18°C, the pH was raised to 7.4, and 1 mL of 10 mM all-trans retinal in EtOH added along with isotopically labeled nutrients 10 minutes before induction of protein expression with 1 mM IPTG. The isotopically labeled nutrients included 1 g of ^2H-D-glucose [Sigma-Aldrich], 1 g of $^{15}N/^2H$ algal-derived amino acids [Martek] depleted of isoleucine, leucine, valine, methionine, and tyrosine, 60 mg α-ketobutyrate [Sigma-Aldrich] and 100 mg α-ketoisovalerate [Cambridge Isotope Laboratories] both with $^{13}CH_3$ methyl labeling of one isotopomer and ^{12}CD labeling elsewhere, 50 mg phenyl-deuterated L-tyrosine [Sigma Aldrich], 50 mg $^{13}CH_3/^{12}CD$ L-methionine [Cambridge Isotope Laboratories]; 0.24 g $MgSO_4$ and 15 mg $CaCl_2$ were added as well. The algal-derived amino acids were depleted of I, L, V and M (and Y) amino acids by reverse-phase HPLC; appropriately isotopically labeled precursors for ILV were then used along with labeled methionine and tyrosine as described above. Protein was expressed for ~3 hours at 18°C before glucose was fully depleted, with 1 mL of 10 mM all-trans retinal added every hour (30 μM total final concentration). Cells were spun down and frozen. Further details of this expression strategy are described elsewhere [1] This quantity of $^2H/^{15}N$ amino acids results in ~80% final ^2H incorporation.

Purification of pSRII - pSRII was purified in a slightly different manner to that described previously [2] Cell pellets (1 L culture) were resuspended in 20 mL 100 mM Tris-HCl pH 7.4, 400 mM NaCl and 20% glycerol. Hen egg-white lysozyme (20 mg), DNAase (4 mg), 800 μL 1 M $MgCl_2$, 0.6 g dodecyl-maltopyranoside (DDM), and 2 EDTA-free protease inhibitor tablets [COEDTAF-RO Roche] were then added. The mixture was diluted to 40 mL with deionized H_2O and rotary mixed in the dark overnight. The lysate was then centrifuged at 32,000 g for 45 minutes at 4°C. The insoluble pellet was discarded and 1 M imidazole was added to the supernatant to a final concentration of 20 mM (final volume ~ 40 mL) which was loaded at 0.7 mL/min onto a nickel

column pre-equilibrated with 50 mM phosphate buffer pH 7.4, 150 mM NaCl, 10% glycerol, 20 mM imidazole, and 0.1% DDM detergent. The column was washed with 5 column volumes of the same buffer followed by five column volumes of the buffer supplemented with 750 mM NaCl and 50 mM imidazole. DDM detergent was exchanged for deuterated d_{26}-diheptanoyl-phosphocholine (DHPC) detergent [CortecNet] as previously described[2b] and then eluted with 0.28% d_{26}-DHPC in pH 6.0 50 mM phosphate, 300 mM imidazole with 50 mM NaCl and 0.02% sodium azide. The protein was exchanged into the same phosphate buffer without imidazole and concentrated to a final protein concentration not exceeding 500 μM.

Expression of Labelled OmpW – OmpW cloning and the plasmid construct used in this study has been previously described [3] Expression of isotopically labeled OmpW was carried out in an HMS *E coli* cell line using the *lac* expression system. A glycerol stock, kept at -80°C, was used to inoculate 3 mL starter growths. These starter cultures were grown in LB media supplemented with 0.1 mg/mL ampicillin at 37°C for 8 to 10 hrs. Cells were pelleted and resuspended in 1 mL of M9 media supplemented with 0.1 mg/mL ampicillin. Isotope incorporation for NMR studies was accomplished using $^{15}NH_4Cl$ (Cambridge Isotopes: NLM-467), uniform ^{2}H or $^{13}C/^{2}H$ glucose (Cambridge Isotopes: DLM-2062, CDLM-3813), and D_2O (Cambridge Isotopes: DLM-4). Selective methyl labeling was accomplished using ketoacid ILV precursors [4] Labeling for protein used in methyl relaxation experiments utilized α-ketobutyrate and α-ketoisovalerate that lead to $^{13}CH_3$ methyl labeling of one isotopomer and ^{12}CD labeling elsewhere (Sigma-Aldrich 589276, Cambridge Isotopes CDLM 7317). Selective side chain labeling for methyl assignment experiments was accomplished using α-ketobutyrate and α-ketoisovalerate that lead to $^{13}CH_3$ methyl labeling of one isotopomer and ^{13}CD labeling elsewhere (Cambridge Isotopes: CDLM-4611, CDLM-8100). Two separate 25 mL growths were inoculated using 500 μL of resuspended cell solution. The 25 mL cultures were grown for ~17 hrs (to an OD between 0.8 and 0.9) and then diluted to 500 mL in M9 media. The 500 mL cultures were grown to an OD of ~0.6 (taking approximately 13 hours). At this point, the ILV precursors were added to the culture. After 1 hour, cells were induced by adding 1 mM IPTG. The induced culture was pelleted after ten hours and then frozen at -20°C.

Purification of OmpW – The procedure for inclusion body purification has been previously detailed for outer membrane proteins [3, 5] and is summarized briefly. The frozen cell pellet was thawed and resuspended in a lysis buffer (100 mM Tris-HCl, pH 8.0, 10 mM EDTA). The cells were then lysed using an Emulsiflex Cell Homogenizer. Following lysis, 87 μL of Brij-35 detergent was added to 25 mL of lysed cells. The lysed cell solution was then centrifuged at ~4,500 G for 20 minutes. The supernatant was discarded and the pellet resuspended in a wash buffer (100

mM Tris-HCl, pH 8.0, 1 mM EDTA) and centrifuged for an additional 20 minutes. The wash process was repeated, and then the inclusion body pellet was frozen at -20°C. The frozen inclusion body pellet was thawed and resuspended in 8 M urea buffered to pH 8.0 with 20 mM Tris-HCl. Final denatured OmpW concentration was 50 μM. The denatured OmpW was diluted dropwise into refolding buffer at 45°C being stirred at 600 RPM on a desktop stir plate. Final conditions for the refolding reaction were 4 μM OmpW, 20 mM Tris-HCl pH 8.0, and 6.5 mM SB3-12 detergent. After 12 hours, the reaction was quenched by adding acetate buffer to a final concentration of 40 mM and stirred for 1 hour and then filtered using a 0.45-micron syringe filter. The filtered solution was then concentrated to a final volume of ~400 μL using 30 kD spin concentrators (EMD Millipore). Using the same concentrators, the buffer was exchanged into final conditions. Final buffer conditions were 40 mM phosphate buffer, pH 7.0, 6.5 mM SB3-12 detergent, and 10% (volume ratio) D_2O. Final sample volume was 370 μL and concentration was assessed by absorbance at 280 nm and calculated using a molar extinction coefficient of 39,420 M^{-1}. NMR sample was contained in a Shigemi tube (Sigma-Aldrich). Final protein concentrations for NMR samples were 275 μM (relaxation) and 670 μM (assignment).

pSRII & OmpW Bicelle Incorporation – Rather than exchange into d_{26}-c_7-DHPC detergent, for bicelles pSRII was similarly exchanged on-resin for 20 mM 1,2-dihexanoyl-sn-glycero-3-phosphocholine (c_6-DHPC). pSRII in deuterated c_6-DHPC micelles was eluted with buffer containing additional (20 mM) c_6-DHPC and 300 mM imidazole. Eluted protein was buffer exchanged into the pSRII NMR buffer to remove imidazole. ^{1}H-NMR was used to confirm the removal of DDM. The long-chain lipid was prepared for bicelle incorporation by dissolving deuterated d_{54}-DMPC to 50 mg/mL in chloroform. The amount needed to construct a sample of 1:1 DMPC:c_6-DHPC (q=1.0) was added to a glass vial. A nitrogen stream was used to dry the DMPC into a thin layer against the glass. pSRII in c_6-DHPC micelles was added to the dried lipid and vortexed. Five freeze-thaw cycles were performed by moving the sample between ice and a 42°C water bath in 5-minute intervals to further clarify the sample. NMR and SAXS experiments were performed as outlined below.

OmpW-containing bicelles were generated by resuspending 1,2-dimyristoyl-sn-glycero-3-phosphocholine (DMPC) in deuterated c_6-DHPC-solubilized OmpW in pH 10, 40 mM borate buffer. DMPC was resuspended to a final bicelle q-value of 1.0 by performing 5 cycles of vortexing, heating to 42°C for 3 minutes, and chilling on ice for 3 minutes. ^{15}N-TROSY spectra used in Fig. S1 were collected for OmpW in SB3-12 micelles at pH 10 with 40 mM borate buffer for comparison with bicelle-incorporated OmpW in order to have identical buffer conditions.

MBP expression & purification – Deuterated, ^{15}N-labeled maltose binding protein, with $^{13}CH_3$ labeled isoleucine, leucine, valine, and methionine residues was grown and purified as previously described [6]. The same isotopic labeling strategy and scheme as for OmpW above was used for MBP.

Small-angle X-ray Scattering

pSRII and OmpW embedded in bicelles with q = 1.0 were used to construct a series of varying q ratios. NMR sample conditions were employed. The short-chain proportion was increased by adding c_6-DHPC in the appropriate amount. The following ratios were measured via SAXS: q = 1.0, 0.8, 0.6, 0.4, 0.2. SAXS measurements were conducted using the 16ID-LiX Beamline at the National Synchrotron Light Source II, located at the Brookhaven National Laboratory (Upton, NY) [7] Data were collected with the standard flow-cell-based solution scattering setup and at a wavelength of 1.0 Å in a three-camera configuration, yielding accessible scattering angles where $0.005 < Q < 3.0$ Å-1, where q is the momentum transfer, defined as $Q = 4\pi \sin(\theta)/\lambda$, where λ is the X-ray wavelength and 2θ is the scattering angle. Radial averaging and Q-conversion of data were performed using beamline-specific software, merging data from all three detectors used in the measurements. Transmission correction and background subtraction were performed to minimize the intensity of the hydrogen bond from water at ~2.0 Å^{-1}. 45 µL of sample at the same concentration used for NMR experiments was exposed for 5 replicates of 1-s each and averaged.

NMR Spectroscopy

Micelle-incorporated pSRII – All experiments were collected at 50 °C on Bruker AVANCE III spectrometers equipped with TCI cryoprobes. Most experiments were carried out at 750 MHz (^{1}H). Assignments for the backbone amide and side chain methyl groups (methionine Cε, leucine Cδ1 and Cδ2, valine Cγ1 and Cγ2, and isoleucine Cδ1) were mapped from previous studies [2b, 8] and confirmed with a 3D NOESY ^{1}H-^{15}N TROSY experiment (backbone amide) and a 3D NOESY ^{1}H-^{13}C HMQC experiment (side chain methyl). These experiments were collected with non-uniform sampling at a sampling density of 15% with a Poisson-gap distribution [9] Correlation with previous assignments are excellent ($R^2 > 0.996$). Two-dimensional ^{13}C-HMQC and ^{15}N-TROSY experiments were collected with 200 and 128 complex indirect points, respectively. A 3D NOESY – ^{13}C,^{1}H – HSQC experiment with 20% sampling density with a Poisson gap distribution and 200 ms mixing time was collected to observe the presence of specific methyl-water interactions in the core of the protein. All non-uniformly sampled data were reconstructed with iterative soft thresholding (istHMS)[9] and processed in nmrPipe[10].

^{15}N-TROSY-sampled ^{15}N T_1 relaxation experiments[11] for the micelle-incorporated pSRII were collected at 750 MHz with relaxation delays of 0.000, 0.320 (2x), 0.560, 0.880, 1.200 (2x), and 1.600 and at 600 MHz with relaxation delays of 0.000, 0.320 (2x), 0.640, 0.960, 1.440 (2x), and 2.000 s. ^{15}N-TROSY sampled $T_{1\rho}$ relaxation experiments[11] were collected at 750 MHz with relaxation delays of 0.001, 0.0077 (2x), 0.0141, 0.0218, 0.0304 (2x), and 0.0400 s at 600 MHz with relaxation delays of 0.001, 0.0067 (2x), 0.0124, 0.0190, 0.0266 (2x), and 0.035 s. Decay curves for resolved peaks in the ^{15}N-TROSY spectrum were fitted to single-exponential decays with errors determined by deviations between duplicate delay values. $T_{1\rho}$ values were converted to their corresponding T_2 values using the ^{15}N spin-lock RF field strength (ω_1) and the individual offset from the ^{15}N carrier in Hz (Ω) for each peak using Eq. 1 where $\tan\theta = \omega_1/\Omega$ [11]

$$R_2 = R_{1\rho} / \sin^2\theta - R_1 / \tan^2\theta \qquad \textbf{(1)}$$

The obtained T_1 and T_2 values were used to determine the effective tumbling times for each residue as calculated [12]:

$$\tau_c = \frac{1}{4\pi\upsilon_N}\sqrt{6\frac{T_1}{T_2} - 7} \qquad \textbf{(2)}$$

After excluding outliers, largely contributed by locally dynamic regions, from the distribution (see Fig. S3), the average was selected as the global reorientation time yielding values of 22.8 and 21.0 ns for data obtained at 600 and 750 MHz (^{1}H), respectively. An average tumbling time of 21.9 ± 0.9 ns was used for analysis of cross-correlated relaxation data, which is in very good agreement with a previously published value (21.3 ns) [2a]. As further validation of the global tumbling time, a separately grown, purified, and prepared sample was analyzed in a similar manner, yielding values of 22.7 ns (600 MHz) and 21.3 ns (750 MHz).

Backbone amide [13] and side chain methyl [14] Carr-Purcell-Meiboom-Gill (CPMG) experiments were collected on micelle-incorporated pSRII at 750 MHz with various spacing between adjacent 180° pulses (1.00, 1.25, 1.67, 2.50, 3.33 (2x), 5.00, 6.67, 10.00, 20.00 ms intervals in a 40 ms constant time period for backbone amide CPMG and 1.00, 1.25, 1.67 (2x), 2.00, 2.50, 2.86, 3.33, 4.00, 5.00, 6.66, 10.00, and 20.00 ms spacings in a 20 ms constant time period for side chain methyl CPMG experiments) to detect the presence of intermediate timescale (μs – ms) motions.

Side chain methyl (ILVM) order parameter (O^2_{axis}) values were determined by cross-correlated relaxation experiments [15] Single- and triple-quantum ^{1}H-^{13}C coherence transfer experiments were collected at a series of delay times: 0.003, 0.005, 0.008 (2x), 0.012, 0.017, 0.022 (2x), 0.028, and 0.036 s. More scans (144/FID) were acquired for the triple quantum

experiment than for the single-quantum experiment (64/FID) and the raw peak intensities normalized accordingly. Ratios of normalized peak intensities were fitted for η and δ values using Eq. 3 where T is the relaxation time [15]:

$$\left|\frac{I_a}{I_b}\right| = \frac{3}{4}\frac{\eta \tanh\left(\sqrt{\eta^2+\delta^2}T\right)}{\sqrt{\eta^2+\delta^2} - \delta \tanh\left(\sqrt{\eta^2+\delta^2}T\right)} \quad \textbf{(3)}$$

O^2_{axis} values were then determined for each methyl probe as in Eq. 4, using their fitted η values and the separately determined macromolecular reorientation time (τ_c) of 21.9 ± 0.9 ns. For slow isotropic macromolecular tumbling [15]:

$$\eta \simeq \frac{9}{10}\left(\frac{\mu_0}{4\pi}\right)\left[P_2(\cos\theta_{axis,HH})\right]^2 \frac{O^2_{axis}\gamma_H^4\tau_c}{r_{HH}^6} \quad \textbf{(4)}$$

where μ_0 is the vacuum permittivity constant, γ_H is the proton gyromagnetic ratio, r_{HH} is the distance between protons within a methyl group (1.813 Å), $\theta_{axis,HH}$ is the angle between the methyl symmetry axis and a vector between a pair of methyl protons (90°), and $P_2(x) = ½(3x^2 - 1)$. Uncertainties in the final O^2_{axis} values were determined by 500 Monte Carlo samplings incorporating errors in fitted η values and tumbling time. Fitted η and δ values, χ^2, local reduced χ^2, final O^2_{axis} values and their errors are provided in Table S1, and order parameter values and associated errors are deposited to the BMRB under accession number 27465. A comprehensive set of L-S order parameters were obtained from cross-correlated relaxation experiments at 50 °C. To examine the temperature sensitivity of motion, cross-correlated relaxation experiments were also carried out at 35 °C for the micelle-incorporated receptor. Because of an increased tumbling time (τ_c of 30.2 ± 0.9) at the lower temperature the sensitivity was greatly reduced and only a subset of peaks (~35) in the full spectrum could be analyzed quantitatively for comparison. A Bayesian statistical model [16] where the data are analyzed for the presence of an unknown number (1-5) of motional classes was used to evaluate the distribution of O^2_{axis} values. The 3-banded distribution was further explored with a k-means clustering algorithm described previously [17].

Bicelle-incorporated pSRII – ^{15}N-TROSY sampled ^{15}N T_1 relaxation experiments [11] for the bicelle-incorporated pSRII were collected at 800 MHz with relaxation delays of 0.000, 0.160 (2x), 0.480, 1.280, 1.840 (2x), and 2.400. ^{15}N-TROSY sampled $T_{1\rho}$ relaxation experiments[11] were collected at 800 MHz with relaxation delays of 0.001, 0.002 (2x), 0.006, 0.012, 0.02 (2x), and 0.0300 s. Decay curves for resolved peaks in the ^{15}N-TROSY spectrum were fitted to single-exponential decays with errors determined by deviations between duplicate delay values. $T_{1\rho}$

values were converted to their corresponding T_2 values as above. Tumbling time was calculated as above, with the error being the standard deviation of the individual site tumbling time distribution.

Single- and triple-quantum ^{1}H-^{13}C coherence transfer experiments were collected at a series of delay times: 0.002, 0.003, 0.005 (2x), 0.007, 0.010, 0.013, 0.016 (2x), 0.019, and 0.022 s. More scans (144/FID) were acquired for the triple quantum experiment than for the single-quantum experiment (32/FID) and the raw peak intensities normalized accordingly. Side chain methyl order parameter values were calculated as above for the micelle-incorporated pSRII.

Micelle-incorporated OmpW – ^{15}N-TROSY sampled ^{15}N T_1 relaxation experiments [11] were collected at 600 MHz with relaxation delays of 0.000, 0.320 (2x), 0.640, 0.960, 1.440 (2x), 1.920, and 2.400 s. ^{15}N-TROSY sampled $T_{1\rho}$ relaxation experiments [11] were also collected at 600 MHz with relaxation delays of 0.001, 0.0067 (2x), 0.0124, 0.0190, 0.0266 (2x), and 0.0350 s Decay curves for resolved peaks in the ^{15}N-TROSY spectrum analyzed identically. Single- and triple-quantum ^{1}H-^{13}C coherence transfer experiments were collected at a series of delay times: 0.001, 0.003, 0.005 (2x), 0.007, 0.010, 0.013, 0.016, 0.019 (2x), 0.022, and 0.026 s. More scans (96/FID) were acquired for the triple quantum experiment than for the single-quantum experiment (32/FID) and the raw peak intensities normalized accordingly. Ratios of normalized peak intensities were fitted for η and δ values as described for pSRII above.

Bicelle-incorporated OmpW – ^{15}N-TROSY sampled ^{15}N T_1 relaxation experiments [11] were collected at 800 MHz with relaxation delays of 0.000, 0.160 (2x), 0.480, 1.280, 1.840 (2x), and 2.400 s. ^{15}N-TROSY sampled $T_{1\rho}$ relaxation experiments were also collected at 800 MHz with relaxation delays of 0.001, 0.002 (2x), 0.004, 0.008, 0.012, 0.016 (2x), 0.020, and 0.025 s. Decay curves for resolved peaks in the ^{15}N-TROSY spectrum were analyzed identically. Single- and triple-quantum ^{1}H-^{13}C coherence transfer experiments were collected at a series of delay times: 0.002, 0.003, 0.005 (2x), 0.007, 0.010, 0.013, 0.016 (2x), 0.019, and 0.022 s. More scans (96/FID) were acquired for the triple quantum experiment than for the single-quantum experiment (32/FID) and the raw peak intensities normalized accordingly. Ratios of normalized peak intensities were fitted for η and δ values as described for pSRII above.

Maltose binding protein – ^{15}N-TROSY sampled ^{15}N T_1 relaxation experiments [11] were collected at 750 MHz with relaxation delays of 0.000, 0.240 (2x), 0.400, 0.560, 0.800 (2x), 1.040, 1.360, 1.680 (2x), and 2.000 s. ^{15}N-TROSY-sampled $T_{1\rho}$ relaxation experiments were also collected at 750 MHz with relaxation delays of 0.0010, 0.0058 (2x), 0.0106, 0.0163, 0.0228 (2x), 0.0300, 0.0380, 0.0460, and 0.0550 s. Decay curves for resolved peaks in the ^{15}N-TROSY spectrum were analyzed identically. The global reorientation time for MBP in H_2O buffer were

determined as for pSRII above (16.2 ± 0.7 ns). Single- and triple-quantum ^{1}H-^{13}C coherence transfer experiments were also collected at 750 MHz at a series of delay times: 0.001, 0.004, 0.008 (2x), 0.012, 0.016, 0.020, 0.024 (2x), 0.028, 0.032, and 0.034 s. More scans (48/FID) were acquired for the triple quantum experiment than for the single-quantum experiment (16/FID) and the raw peak intensities normalized accordingly. Ratios of normalized peak intensities were fitted for η and δ values as described for pSRII above. Methyl side chain order parameters were calculated as above for pSRII; however, because the experiments were collected in D_2O buffer, the global reorientation time was multiplied by 1.223 as to account for the known viscosity difference in the solvents.

Molecular dynamics simulations

Hydrogens were add to the structure of pSRII (PDB:1H68)[18] with VMD [19] and parameters/topology for the covalently attached retinal cofactor (Lys205) were used as deposited in the NAMD [20] repository. The protein, retinal, and its structural water molecules were inserted into an 8.0 by 8.0 nm bilayer composed of 1-palmitoyl-2-oleoyl-sn-glycero-3-phosphocholine (POPC) generated in VMD to maintain nonpolar and aromatic molecules in the bilayer [19] The protein-containing bilayer was then hydrated with an additional 8 Å of TIP3P water [21] on either side along with the removal of any lipid molecules overlapping the protein. All other aspects of the simulation were as previously described [22] for 1000 steps of minimization, 200 ps of equilibration, and 100 total ns of simulation at 50 °C (as in experiment).

Two molecular dynamics simulations of OmpW (PDB:2MHL [23]) (one in a bilayer and one in a detergent micelle) were built using the CHARMM-GUI server [24] The bilayer system was built using 1,2-dimyristoyl-sn-glycero-3-phosphocholine (DMPC). DMPC is well characterized by simulations and is known to match the hydrophobic thickness of the native outer membrane [25] The micelle system was built using the SB3-12 detergent.

Simulation systems were neutralized using 150 mM NaCl and under NPT conditions (40°C and 1 atm, as in experiment). Simulations were run using NAMD[20] employing the CHARMM36 forcefield[25a] with 2.0 femtosecond (fs) time steps. The standard equilibration protocol provided by CHARMM-GUI was followed. Following equilibration, the first 50 ns of simulation were used to equilibrate the bilayer or micelle atoms. Simulation temperature was controlled using the Nose-Hoover thermostat, with a damping coefficient of 1.0 ps^{-1}, and the bath was not coupled to hydrogen atoms. Pressure was controlled using the Langevin piston with a constant pressure piston of 50 fs and decay time equal to 25 fs. Long range electrostatics were evaluated using the Particle Mesh Ewald summation [26] Both short range electrostatics and Leonard Jones interactions were cutoff at 12 Å, with a smooth switching function applied beginning at 10 Å. Non-

bonded pairs were determined using a distance cutoff of 16 Å, and the list was updated every 20 femtoseconds. Long range and electrostatic interactions were evaluated every step. The final trajectories were run for 300 ns.

Backbone O^2 and side chain methyl O^2_{axis} values were calculated as previously described[22b] using the long-time limit approximation. All 100 ns of pSRII simulation time was included in the calculation (~5x τ_c) while the 250 ns of simulation for OmpW was split into two separate 125 ns (also ~5x τ_c) trajectories for analysis.

Structural images were prepared with PyMol (Schrödinger).

Table S1. Methyl relaxation data and determined O^2_{axis} parameters for pSRII in DHPC micelles at 50 °C at 750 MHz (^{1}H) (deposited to the BMRB under accession number 27465).

Atom	δ[a]	η[a]	η error[b]	Reduced $\chi^{2[c,d]}$	O^2_{axis} [d]	O^2_{axis} error [e]
Met1 Cε	0.30	2.65	0.37	1.34	0.034	0.005
Val2 Cγ1	-4.23	7.64	0.58	0.46	0.097	0.008
Val2 Cγ2	-16.17	8.81	0.95	0.30	0.112	0.013
Leu4 Cδ1	-25.19	16.31	3.37	1.11	0.206	0.044
Leu4 Cδ2	-13.39	16.08	0.63	0.13	0.203	0.011
Leu7 Cδ2	-36.18	23.89	1.68	0.44	0.302	0.024
Leu10 Cδ1	-10.72	13.30	0.90	1.04	0.168	0.013
Ile13 Cδ1	-16.07	23.75	0.82	2.31	0.301	0.016
Leu16 Cδ1	-8.90	13.33	0.93	1.14	0.169	0.014
Leu20 Cδ1	-20.82	22.80	1.83	1.60	0.288	0.025
Val38 Cγ1	-17.53	30.65	1.11	0.70	0.388	0.022
Val38 Cγ2	-32.28	32.71	3.09	1.42	0.414	0.042
Leu40 Cδ1	-22.29	33.16	9.81	0.20	0.420	0.119*
Leu40 Cδ2	-43.24	34.64	7.59	1.59	0.438	0.099
Ile46 Cδ1	-38.72	36.41	3.52	2.22	0.461	0.050
Val49 Cγ2	-54.83	54.05	3.98	0.77	0.684	0.057
Val53 Cγ1	-31.64	38.79	3.79	2.75	0.491	0.051
Val53 Cγ2	-31.81	37.25	3.13	0.76	0.471	0.042
Leu56 Cδ1	-17.34	12.38	1.53	0.98	0.157	0.021
Leu56 Cδ2	-44.44	39.98	5.22	1.91	0.506	0.069
Val58 Cγ1	-26.55	42.66	3.20	1.29	0.540	0.048
Val58 Cγ2	-35.83	47.96	1.53	0.80	0.607	0.033
Val61 Cγ1	-32.57	32.44	3.57	0.73	0.411	0.050
Val63 Cγ1	-27.93	35.26	2.01	0.25	0.446	0.031
Val63 Cγ2	-61.96	42.48	3.29	2.00	0.538	0.047
Val68 Cγ1	-36.02	47.73	3.85	3.58	0.604	0.054
Ile74 Cδ1	-16.88	24.48	0.73	1.08	0.310	0.016
Ile77 Cδ1	-43.39	44.02	1.95	0.96	0.557	0.033
Leu78 Cδ1	-49.62	44.75	4.42	0.89	0.566	0.060

Leu78 Cδ2	-32.17	33.68	3.46	0.82	0.426	0.048
Val84 Cγ1	-41.46	39.65	3.91	1.01	0.502	0.054
Val84 Cγ2	-51.89	38.15	3.14	0.83	0.483	0.046
Leu87 Cδ1	-37.55	39.63	2.32	0.53	0.502	0.036
Leu89 Cδ1	-27.41	28.52	2.14	0.36	0.361	0.031
Leu89 Cδ2	-31.87	29.99	3.00	0.39	0.380	0.041
Leu90 Cδ1	-26.21	23.22	2.50	0.66	0.294	0.035
Leu90 Cδ2	-28.50	26.16	3.79	0.94	0.331	0.050
Leu93 Cδ1	-35.44	26.49	3.94	0.81	0.335	0.050
Leu93 Cδ2	-33.92	24.98	4.25	0.96	0.316	0.058
Ile100 Cδ1	-21.06	29.84	1.23	1.61	0.378	0.023
Val101 Cγ1	-18.16	14.05	1.26	0.60	0.178	0.018
Val101 Cγ2	-24.17	14.31	2.03	1.22	0.181	0.025
Ile102 Cδ1	-17.06	13.40	0.56	0.65	0.170	0.010
Val107 Cγ1	-51.98	55.85	3.30	0.68	0.707	0.051
Met109 Cε	-82.07	53.77	7.27	0.55	0.680	0.099
Met117 Cε	-4.03	13.57	0.21	0.16	0.172	0.008
Val118 Cγ1	-40.94	41.54	4.45	1.30	0.526	0.062
Ile121 Cδ1	-4.51	14.99	0.49	2.18	0.190	0.010
Met129 Cε	-2.75	5.38	0.19	0.47	0.068	0.004
Ile135 Cδ1	-21.84	25.25	1.46	1.72	0.320	0.023
Leu137 Cδ1	-38.96	29.25	4.00	0.61	0.370	0.054
Leu137 Cδ2	-37.35	25.39	5.75	1.99	0.321	0.072
Val138 Cγ1	-55.28	43.65	4.85	0.41	0.552	0.068
Val138 Cγ2	-45.71	48.69	3.45	0.78	0.616	0.050
Leu141 Cδ1	-27.69	23.51	3.37	0.89	0.298	0.046
Leu141 Cδ2	-9.02	14.74	0.46	0.58	0.187	0.010
Val142 Cγ2	-25.83	27.00	2.84	0.73	0.342	0.040
Met145 Cε	-39.95	29.90	3.95	1.32	0.378	0.051
Ile156 Cδ1	-16.59	16.69	0.62	0.74	0.211	0.012
Val161 Cγ1	-36.85	44.82	4.38	0.92	0.567	0.061
Val161 Cγ2	-46.86	40.60	3.61	0.46	0.514	0.049
Val163 Cδ2	-50.51	36.08	6.45	0.64	0.457	0.078
Leu166 Cδ2	-34.01	16.80	2.78	0.77	0.213	0.039

Leu166 Cδ1	-19.04	26.57	1.89	1.42	0.336	0.028
Val168 Cγ1	-30.94	39.89	2.84	1.20	0.505	0.040
Leu170 Cδ2	-7.90	12.75	0.99	0.39	0.161	0.015
Ile173 Cδ1	-13.47	23.61	0.80	2.70	0.299	0.016
Ile177 Cδ1	-21.01	17.22	1.14	0.70	0.218	0.017
Leu179 Cδ1	-9.93	16.88	1.43	0.90	0.214	0.020
Leu179 Cδ2	-14.36	15.63	1.44	0.97	0.198	0.020
Val185 Cγ1	-21.51	29.13	1.34	0.34	0.369	0.025
Val185 Cγ2	-47.29	27.46	5.75	0.89	0.347	0.075
Leu187 Cδ1	-7.26	14.68	0.58	0.37	0.186	0.011
Leu187 Cδ2	-6.09	10.43	0.71	0.90	0.132	0.011
Leu188 Cδ2	-45.87	42.60	6.46	1.76	0.539	0.081
Val192 Cγ2	-62.96	52.54	3.04	0.15	0.665	0.048
Val194 Cγ1	-82.05	69.48	8.41	0.03	0.879	0.113*
Leu196 Cδ1	-33.12	32.27	2.88	2.88	0.408	0.041
Leu196 Cδ2	-34.89	34.26	6.13	2.80	0.434	0.080
Ile197 Cδ1	-16.90	10.85	1.35	0.83	0.137	0.018
Val198 Cγ1	-37.19	36.38	3.30	0.79	0.460	0.045
Leu200 Cδ1	-72.41	38.26	6.89	0.56	0.484	0.092
Leu202 Cδ2	-35.26	34.79	4.11	1.40	0.440	0.054
Val203 Cγ1	-33.56	38.91	5.14	1.53	0.492	0.066
Val203 Cγ2	-43.20	38.91	2.90	1.12	0.492	0.045
Val206 Cγ1	-41.09	48.94	6.92	2.50	0.619	0.094
Ile211 Cδ1	-33.43	35.51	1.73	1.36	0.449	0.029
Leu213 Cδ1	-21.88	20.83	1.30	0.53	0.264	0.020
Leu213 Cδ2	-17.78	17.91	1.70	1.35	0.227	0.024
Leu219 Cδ2	-10.78	16.50	1.71	1.60	0.209	0.024
Val230 Cγ1	-12.60	1.80	0.20	0.55	0.023	0.003
Val237 Cγ1	-12.02	1.74	0.25	0.92	0.022	0.003

[a] η and δ were fitted using Eq. 1. [b] Errors in η were estimated from the covariance in fitting to Eq. 1 and incorporated the reduced χ^2 values. [c] Reduced χ^2 for the fit with Eq. 1. [d] O^2_{axis} values were determined from Eq. 2 using the fitted η values and the calculated tumbling molecular reorientation time (21.9 ns for the data in this Table). [e] Errors in final O^2_{axis} were determined by

Monte Carlo simulations incorporating errors in fitted η as well as the error in the determined reorientation time.

*methyl groups denoted with an asterisk are not included in detailed statistics in Fig. 2 due to the error in O^2_{axis} being greater than ±0.10.

Table S2. Methyl relaxation data and determined O^2_{axis} parameters for OmpW in SB3-12 micelles at 40 °C at 600 MHz (^{1}H).

Peak Number	δ[a]	η[a]	η error[b]	Reduced χ^{2}[c,d]	O^2_{axis} [d]	O^2_{axis} error [e]
a1	-13.17	20.74	0.26	0.46	0.238	0.008
a2	-12.24	22.02	0.45	1.11	0.253	0.010
a3	-15.31	16.84	0.59	2.34	0.193	0.010
a4	-9.36	22.50	0.41	1.18	0.258	0.010
a5	-13.14	24.25	0.44	0.94	0.279	0.010
a6	-11.12	24.42	0.61	0.97	0.281	0.011
a7	-13.04	24.18	0.39	0.58	0.278	0.010
a8	-14.26	19.20	0.67	0.95	0.221	0.011
a9	-3.94	7.45	0.34	1.79	0.086	0.005
a10	-5.42	10.55	0.47	1.32	0.121	0.007
a11	-33.89	42.63	2.14	1.64	0.490	0.029
a12	-8.91	8.90	0.15	0.49	0.102	0.004
a13	-35.02	48.87	1.57	1.11	0.561	0.026
a14	-42.51	58.48	3.44	2.21	0.672	0.049
a15	-24.27	38.34	1.35	1.10	0.440	0.021
a16	-28.84	38.04	1.02	0.45	0.437	0.019
a17	-49.49	66.86	2.98	1.12	0.768	0.041
a19	-6.21	13.42	0.40	0.89	0.154	0.007
a20	-21.90	34.47	0.90	0.70	0.396	0.017
a21	-31.34	38.11	0.96	0.67	0.438	0.018
a23	-31.54	63.07	2.48	0.63	0.725	0.037
a25	-32.07	57.52	1.51	0.55	0.661	0.028
a26	-34.61	59.96	1.86	1.00	0.689	0.032
a27	-12.79	23.18	0.34	0.24	0.266	0.010
a28	-14.17	21.60	0.40	0.22	0.248	0.009
a29	-23.33	43.35	2.10	0.95	0.498	0.029
a31	-35.61	55.43	1.45	0.66	0.637	0.027
a33	-15.49	32.39	1.59	1.46	0.372	0.021
a34	-45.31	48.46	1.68	0.48	0.557	0.026

a36	-10.47	26.07	1.22	0.68	0.299	0.018
a38	-14.31	41.41	0.41	0.44	0.476	0.017
a40	-23.64	23.87	0.43	1.08	0.274	0.011
a41	-8.37	13.01	0.20	1.02	0.149	0.005
a43	-19.70	35.51	0.63	0.91	0.408	0.015
a44	-9.94	15.12	0.54	4.47	0.174	0.009
a45	-29.46	55.21	1.97	1.96	0.634	0.033
a47	-6.10	17.68	0.27	0.78	0.203	0.007
a48	-9.67	28.29	1.69	18.03	0.325	0.023
a49	-14.11	37.66	0.48	1.87	0.433	0.015
a50	-9.41	28.09	0.23	0.24	0.323	0.011
a51	-10.64	26.52	0.51	1.14	0.305	0.012
a52	-17.58	34.18	0.96	1.37	0.393	0.016
a59	-14.70	33.70	1.06	0.45	0.387	0.017
a60	-9.58	12.19	0.48	3.67	0.140	0.007
a61	-8.34	28.40	2.25	1.05	0.326	0.030
a62	-20.63	36.69	0.65	0.70	0.421	0.017
a100	-31.11	58.20	1.34	2.87	0.669	0.027
a101	-7.45	18.56	0.16	0.56	0.213	0.007
a103	-11.40	22.20	0.88	5.70	0.255	0.014
a104	-8.59	16.71	0.39	0.40	0.192	0.007
a105	-14.58	10.19	0.71	1.08	0.117	0.009
a107	-9.75	15.98	0.39	0.74	0.184	0.008

[a] η and δ were fitted using Eq. 1. [b] Errors in η were estimated from the covariance in fitting to Eq. 1 and incorporated the reduced χ^2 values. [c] Reduced χ^2 for the fit with Eq. 1. [d] O^2_{axis} values were determined from Eq. 2 using the fitted η values and the calculated tumbling molecular reorientation time (24.0 ns for the data in this Table). [e] Errors in final O^2_{axis} were determined by Monte Carlo simulations incorporating errors in fitted η as well as the error in the determined reorientation time.

Table S3: Methyl relaxation data and determined O^2_{axis} parameters for pSRII in DMPC/DHPC bicelles at 50 °C at 800 MHz (^{1}H)

Atom	δ[a]	η[a]	η error[b]	Reduced $\chi^{2[c,d]}$	O^2_{axis} [d]	O^2_{axis} error [e]
Met1 Cε	-44.38	5.07	1.42	0.33	0.049	0.013
Val2 Cγ1	-76.63	38.85	10.81	0.86	0.372	0.099
Leu7 Cδ2	-57.96	54.01	6.26	0.27	0.517	0.060
Ile13 Cδ1	-26.22	37.82	0.98	0.29	0.362	0.011
Met15 Cε	-141.7	78.50	15.63	0.20	0.752	0.149
Leu20 Cδ1	-8.59	25.59	1.52	0.17	0.245	0.015
Val38 Cγ2	-39.66	44.39	5.75	0.72	0.425	0.054
Ile43 Cδ1	-95.25	61.97	4.22	0.05	0.594	0.044
Ile46 Cδ1	-62.34	64.72	5.35	0.69	0.620	0.052
Val49 Cγ2	-39.40	75.31	5.01	0.24	0.721	0.052
Val53 Cγ2	-53.87	78.34	14.65	0.75	0.751	0.137
Leu56 Cδ2	-25.08	48.43	5.95	0.48	0.464	0.060
Val58 Cγ1	-86.65	68.71	15.92	0.34	0.658	0.156
Val58 Cγ2	-51.52	74.91	5.27	0.44	0.718	0.049
Val61 Cγ1	-48.88	55.66	9.67	0.51	0.533	0.091
Val63 Cγ1	-16.96	40.86	7.40	0.32	0.391	0.073
Ile77 Cδ1	-43.94	58.40	2.95	0.46	0.559	0.030
Leu78 Cδ2	-51.07	67.83	9.99	0.70	0.650	0.095
Val84 Cγ1	-32.82	46.60	8.52	1.38	0.446	0.081
Val84 Cγ2	-59.51	58.07	5.58	0.28	0.556	0.054
Leu87 Cδ1	-38.49	62.09	7.71	0.51	0.595	0.072
Leu89 Cδ1	-36.36	41.85	5.10	0.17	0.401	0.049
Leu89 Cδ2	-66.20	55.71	13.46	0.69	0.534	0.125
Leu93 Cδ1	-55.40	49.70	10.59	0.23	0.476	0.105
Ile100 Cδ1	-33.04	48.81	2.11	0.54	0.468	0.020
Ile102 Cδ1	-20.78	21.15	1.39	0.34	0.203	0.013
Met109 Cε	-136.0	76.82	18.42	0.72	0.736	0.180
Met117 Cε	-2.89	17.03	1.13	0.60	0.163	0.011
Ile121 Cδ1	-10.60	24.17	0.50	0.49	0.232	0.006
Met129 Cε	-9.60	9.25	0.68	1.06	0.089	0.007

Ile135 Cδ1	-28.05	37.34	1.38	0.25	0.358	0.014
Leu137 Cδ2	-55.71	52.58	10.66	0.29	0.504	0.106
Val138 Cγ1	-76.86	83.22	9.85	0.22	0.797	0.089
Val138 Cγ2	-57.22	74.50	6.61	0.51	0.714	0.066
Leu141 Cδ1	-30.20	37.54	5.32	0.25	0.360	0.052
Leu141 Cδ2	-11.80	21.82	1.98	0.34	0.209	0.020
Val142 Cγ2	-37.16	37.81	6.31	0.51	0.362	0.062
Met145 Cε	-64.12	48.40	5.82	0.36	0.464	0.058
Ile156 Cδ1	-26.15	25.77	2.25	0.82	0.247	0.022
Val161 Cγ2	-61.09	65.47	5.89	0.11	0.627	0.058
Val163 Cγ2	-50.69	55.60	12.59	0.21	0.533	0.126
Val168 Cγ1	-32.77	53.80	4.52	0.42	0.515	0.045
Leu170 Cδ2	-146.5	42.00	22.63	0.605	0.402	0.238*
Ile173 Cδ1	-24.92	35.62	0.95	0.50	0.341	0.011
Leu179 Cδ1	-15.40	25.38	3.59	0.41	0.243	0.033
Leu179 Cδ2	-0.11	18.73	2.58	0.31	0.179	0.025
Val185 Cγ1	-29.39	43.03	4.82	0.71	0.412	0.047
Val185 Cγ2	-31.34	36.82	9.71	0.46	0.353	0.090
Leu187 Cδ1	-0.23	17.99	1.47	0.21	0.172	0.015
Leu187 Cδ2	-14.90	15.76	2.00	0.32	0.151	0.019
Leu188 Cδ2	-73.68	66.96	10.03	0.38	0.641	0.096
Val192 Cγ2	-105.2	73.49	11.80	0.21	0.704	0.113
Leu196 Cδ1	-57.96	54.01	6.26	0.27	0.517	0.056
Leu196 Cδ2	-31.50	37.59	3.92	0.10	0.360	0.037
Ile197 Cδ1	-23.15	17.06	2.00	0.19	0.163	0.019
Leu202 Cδ2	-52.44	63.23	11.58	0.36	0.606	0.113
Ile211 Cδ1	-59.71	62.48	2.82	0.24	0.599	0.029
Leu213 Cδ2	-39.78	23.49	4.61	0.61	0.225	0.042
Leu219 Cδ2	-26.24	32.23	4.42	0.25	0.309	0.044

[a] η and δ were fitted using Eq. 1. [b] Errors in η were estimated from the covariance in fitting to Eq. 1 and incorporated the reduced χ^2 values. [c] Reduced χ^2 for the fit with Eq. 1. [d] O^2_{axis} values were determined from Eq. 2 using the fitted η values and the calculated tumbling molecular reorientation time (28.9 ns for the data in this Table). [e] Errors in final O^2_{axis} were determined by

Monte Carlo simulations incorporating errors in fitted η as well as the error in the determined reorientation time.

*methyl groups denoted with an asterisk are not included in the correlation in Fig. S5 due to the error in O^2_{axis} being greater than a generous ±0.2 cutoff value. See Fig. S5 legend.

Table S4: Methyl relaxation data and determined O^2_{axis} parameters for OmpW in DMPC/DHPC bicelles at 40 °C at 800 MHz (^{1}H)

Peak Number	δ[a]	η[a]	η error[b]	Reduced $\chi^{2[c,d]}$	O^2_{axis} [d]	O^2_{axis} error [e]
a1	-12.55	33.89	0.54	10.86	0.305	0.011
a2	-12.48	31.05	0.54	9.47	0.279	0.011
a3	-15.16	36.17	0.54	6.14	0.325	0.012
a4	-10.34	35.14	0.53	9.86	0.316	0.012
a5	-15.36	37.96	0.75	12.62	0.341	0.013
a6	-18.16	42.22	0.82	4.37	0.379	0.015
a7	-14.28	39.71	0.68	6.26	0.357	0.014
a8	-14.42	28.81	0.44	3.28	0.259	0.010
a9	-7.00	10.96	0.09	0.99	0.098	0.003
a10	-8.17	11.62	0.20	6.03	0.104	0.004
a11	-43.61	68.78	2.80	2.87	0.618	0.032
a12	-7.16	12.86	0.26	2.77	0.116	0.005
a14	-44.89	85.03	2.13	2.48	0.764	0.031
a15	-35.23	59.86	1.29	1.71	0.538	0.023
a16	-32.78	54.39	1.34	2.52	0.489	0.021
a17	-52.39	100.38	3.23	2.89	0.902	0.043
a18	-58.34	87.24	3.83	3.72	0.784	0.042
a20	-25.36	51.86	1.04	2.36	0.466	0.018
a21	-24.60	48.40	1.04	3.71	0.435	0.018
a22	-47.16	89.70	2.18	0.62	0.806	0.033
a23	-39.82	83.44	1.94	0.82	0.750	0.030
a24	-48.46	80.21	2.78	2.25	0.721	0.033
a25	-37.03	82.22	1.42	0.66	0.739	0.028
a26	-50.03	90.28	2.71	3.44	0.811	0.039
a27	-16.93	28.73	0.38	1.45	0.258	0.009
a28	-16.44	24.71	0.23	0.39	0.222	0.008
a29	-16.13	69.40	1.81	0.70	0.624	0.028
a30	-33.11	54.33	1.54	2.14	0.488	0.021
a31	-40.27	84.61	1.90	3.60	0.760	0.031
a33	-7.49	57.90	1.19	0.81	0.520	0.022

a34	-42.67	65.66	1.69	1.47	0.590	0.024
a36	-16.30	49.19	0.80	0.19	0.442	0.016
a38	-9.58	57.60	1.29	21.16	0.518	0.021
a40	-15.86	41.36	0.31	1.94	0.372	0.013
a41	-3.58	19.61	0.26	2.13	0.176	0.007
a43	-21.01	51.35	1.22	13.17	0.461	0.019
a44	-8.67	24.92	0.55	14.70	0.224	0.009
a45	-35.80	74.53	2.15	7.04	0.670	0.029
a46	-7.81	24.40	0.46	4.73	0.219	0.008
a47	-6.64	27.31	0.44	10.15	0.245	0.009
a48	-10.18	43.83	0.82	14.74	0.394	0.015
a49	-8.51	54.69	0.81	15.25	0.491	0.019
a50	-7.63	40.23	0.61	9.09	0.362	0.014
a51	-6.89	34.42	0.43	3.89	0.309	0.011
a52	-22.76	50.66	0.97	4.47	0.455	0.018
a59	-13.99	45.32	1.14	5.00	0.407	0.018
a60	-3.38	19.48	0.30	2.78	0.175	0.007
a61	-19.27	39.24	1.06	2.56	0.353	0.016
a62	-19.65	54.99	1.27	8.41	0.494	0.021
a100	-27.17	82.57	2.79	22.35	0.742	0.035
a101	-7.26	27.63	0.38	12.14	0.248	0.009
a103	-16.74	26.61	0.39	2.28	0.239	0.009
a104	-9.29	22.98	0.29	2.40	0.207	0.008
a105	-16.22	18.47	0.45	0.72	0.166	0.007
a107	-5.60	28.44	0.58	1.90	0.256	0.011

[a] η and δ were fitted using Eq. 1. [b] Errors in η were estimated from the covariance in fitting to Eq. 1 and incorporated the reduced χ^2 values. [c] Reduced χ^2 for the fit with Eq. 1. [d] O^2_{axis} values were determined from Eq. 2 using the fitted η values and the calculated tumbling molecular reorientation time (29.9 ns for the data in this Table). [e] Errors in final O^2_{axis} were determined by Monte Carlo simulations incorporating errors in fitted η as well as the error in the determined reorientation time.

Table S5: Methyl relaxation data and determined O^2_{axis} parameters for maltose binding protein at 37 °C at 750 MHz (^{1}H)

Atom	δ[a]	η[a]	η error[b]	Reduced χ^{2}[c,d]	O^2_{axis} [d]	O^2_{axis} error [e]
1	-4.50	11.20	0.20	3.15	0.157	0.006
2	-19.45	39.99	0.37	1.26	0.559	0.021
3	-6.72	28.60	0.29	3.42	0.400	0.014
4	-2.88	14.76	0.21	3.74	0.206	0.008
5	-15.24	49.15	1.15	11.70	0.687	0.029
6	-3.28	29.42	1.28	81.35	0.411	0.023
7	-6.66	19.22	0.35	7.83	0.269	0.011
8	-17.10	39.19	0.64	4.84	0.548	0.022
9	-27.32	56.69	1.23	5.12	0.792	0.032
10	-12.87	36.24	0.54	6.15	0.506	0.019
11	-20.03	54.82	1.54	13.83	0.766	0.035
12	-8.97	24.23	0.18	1.39	0.339	0.012
13	-20.39	53.59	1.23	3.45	0.749	0.033
14	-6.43	22.18	0.22	1.62	0.310	0.011
15	-19.43	41.16	0.70	4.45	0.575	0.023
16	-12.80	36.63	0.56	4.57	0.512	0.020
17	-30.88	56.23	1.59	15.87	0.786	0.033
18	-20.20	43.96	0.62	5.51	0.614	0.023
19	-4.91	37.97	1.14	26.54	0.531	0.024
20	-32.21	54.06	1.69	1.08	0.755	0.036
21	-21.12	54.35	1.50	3.54	0.759	0.034
22	-4.50	37.70	1.42	22.82	0.527	0.027
23	-22.05	60.89	1.10	1.92	0.851	0.033
24	0.33	9.60	0.19	2.37	0.134	0.005
25	-22.17	57.61	1.31	3.75	0.805	0.034
26	-4.31	37.61	1.66	39.47	0.526	0.030
27	-18.30	58.99	2.09	5.42	0.824	0.042
28	-13.70	61.10	1.29	3.03	0.854	0.033
29	-15.55	61.01	2.09	8.40	0.853	0.042
30	-16.35	60.72	2.00	7.84	0.848	0.041

31	-15.78	53.34	0.78	1.59	0.745	0.028
32	-7.11	34.30	0.75	3.79	0.479	0.019
33	-18.10	54.69	1.48	5.26	0.764	0.034
34	-11.13	52.43	1.05	3.09	0.733	0.030
35	-22.57	64.43	1.39	1.92	0.900	0.037
36	-4.90	41.43	1.23	5.35	0.579	0.025
37	-16.07	60.28	1.93	7.00	0.842	0.041
38	-11.44	58.24	2.15	8.01	0.814	0.043
39	-13.05	51.16	1.66	5.72	0.715	0.033
40	-15.02	58.86	1.48	3.21	0.823	0.034
41	-42.10	66.17	2.70	5.12	0.925	0.049
42	-8.65	28.60	0.55	3.10	0.400	0.016
44	-13.16	62.73	2.32	8.13	0.877	0.045
46	-6.44	38.25	1.12	14.09	0.535	0.024
47	-18.73	41.88	1.07	2.94	0.585	0.025
48	-19.09	44.48	0.73	1.94	0.622	0.024
49	-5.93	34.08	0.89	10.22	0.476	0.021
50	-15.42	62.02	2.17	12.04	0.867	0.045
51	-16.90	50.33	1.28	4.00	0.703	0.030
52	-30.88	59.84	1.98	2.77	0.836	0.042
53	-8.59	44.00	1.03	6.70	0.615	0.027
54	-26.25	56.35	1.43	2.37	0.788	0.035
55	-24.16	47.76	1.24	1.84	0.667	0.029
56	-9.80	30.08	0.49	2.36	0.420	0.017
57	-22.85	43.34	0.90	1.57	0.606	0.024
58	-29.34	53.46	1.81	3.75	0.747	0.036
59	-13.28	50.72	1.86	8.71	0.709	0.036
60	-13.29	57.41	1.43	5.98	0.802	0.036

[a] η and δ were fitted using Eq. 1. [b] Errors in η were estimated from the covariance in fitting to Eq. 1 and incorporated the reduced χ^2 values. [c] Reduced χ^2 for the fit with Eq. 1. [d] O^2_{axis} values were determined from Eq. 2 using the fitted η values and the calculated tumbling molecular reorientation time (19.8 ns for the data in this Table, calculated from 16.2 ns in H_2O multiplied by 1.223 to account for the viscosity difference in the D_2O buffer used here). [e] Errors in final O^2_{axis}

were determined by Monte Carlo simulations incorporating errors in fitted η as well as the error in the determined reorientation time.

Table S6. Soluble protein average methyl O^2_{axis} values for isoleucine, leucine, valine and methionine residues.[a]

Abbreviation	Protein[ref]	ILVM $\langle O^2_{axis} \rangle$	Temperature (°C)
Ca^{2+}-CaM	Ca^{2+} – saturated calmodulin (CaM) [27]	0.427	35
smMLCK-CaM	CaM – smMLCK(p) complex) [28]	0.478	50
nNOS-CaM	CaM – nNOS(p) complex [29]	0.499	35
ubq	Ubiquitin [17]	0.505	45
smMLCK-CaM	CaM – smMLCK(p) complex [28]	0.532	35
CaMKKa-CaM	CaM – CaMKKa(p) complex [29]	0.544	35
PhoB-DNA	DNA binding domain PhoB – DNA [30]	0.587	37
MBP	Maltose binding protein[b]	0.611	37
MSG	Malate synthase G [31]	0.656	37
Flavodoxin	Bacterial flavodoxin C55A [32]	0.668	35
HEWL	Hen egg-white lysozyme [33]	0.677	35

[a] Source data for Fig. 2

[b] This work (Table S5)

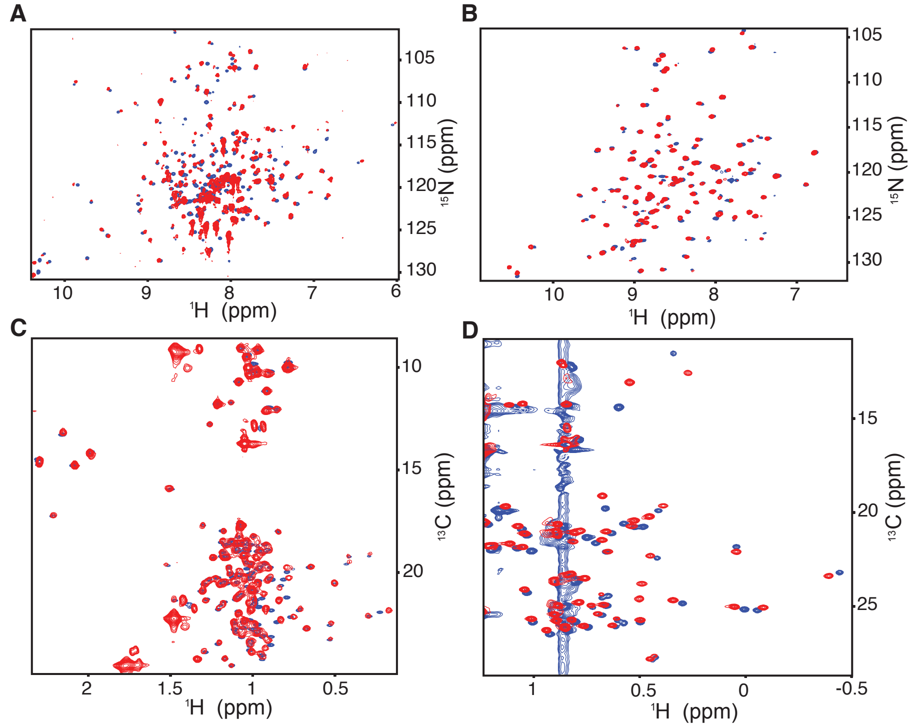

Fig. S1. Comparison of NMR spectra of pSRII & OmpW in micelles and bicelles. Micelle-incorporated spectra are displayed in blue as compared to bicelle-incorporated data in red. pSRII was incorporated into d_{26}-c_7-DHPC micelles and DMPC/c_6-DHPC bicelles (both deuterated) at a q-value of 1.1 while OmpW was incorporated into SB3-12 detergent micelles and DMPC/c_6-DHPC (both deuterated) bicelles, also with a q-value of 1.1. ^{15}N-TROSY spectra of pSRII (50 °C) and OmpW (40 °C) are shown in panels (**A**) & (**B**), respectively, while methyl ^{13}C-^{1}H HMQC spectra of pSRII and OmpW are shown in panels (**C**) & (**D**), respectively. Vertical streaks from detergent molecules are more pronounced in (**D**) for OmpW due to the lack of availability of deuterated SB3-12.

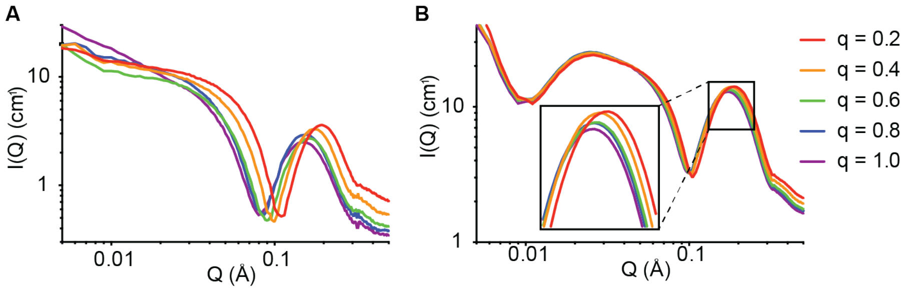

Fig. S2. Small-angle X-ray scattering characterization of bicelle-incorporated membrane proteins at varying q-values. (A) pSRII samples scattered as a function of bicelle q ratio (ratio of DMPC to c_6-DHPC). The q = 1.0 bicelle sample was measured via SAXS (purple). Subsequent samples with lower q-ratios were assembled via addition of c_6-DHPC and measured with the following q ratios: 0.8 (blue), 0.6 (green), 0.4 (orange), 0.2 (red). The change in the prominent peak feature, as observed by Q_{max} (Q value at maximum value) is highlighted with arrows. (**B**) Similarly, OmpW was measured at a series of q-ratios with the same color scheme as for pSRII. Expansion of the major feature of the SAXS profile OmpW diagnosing the transition from bicelle to mixed micelles at q-values below 0.8.

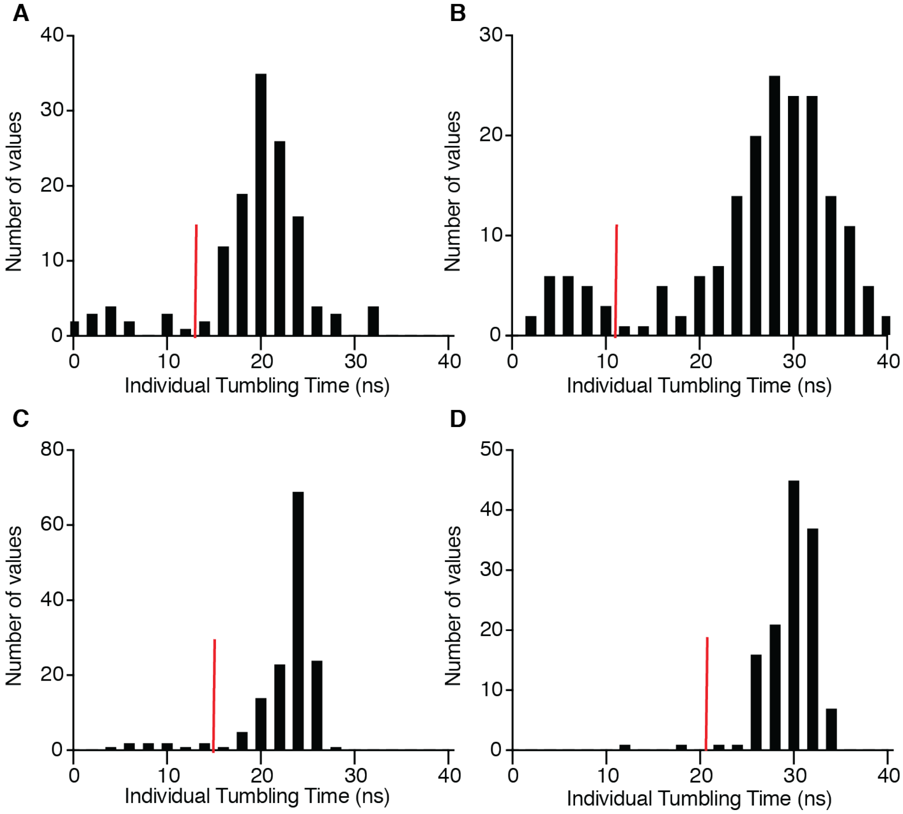

Fig. S3. Distribution of effective tumbling times for pSRII and OmpW. The ^{15}N amide T_1/T_2 ratio at each residue was used to determine local effective tumbling times for pSRII incorporated into detergent micelles (**A**) and bicelles (**B**). Shown here are the distributions obtained using data collected at 750 MHz (^1H) (micelles, **A**) and at 800 MHz (^1H) (bicelles, **B**) for the same sample(s) used subsequently for cross-correlated relaxation experiments. The red lines demarcate outliers discarded before averaging to obtain a global reorientation time. OmpW incorporated into micelles (**C**) and q=1.1 bicelles (**D**) were analyzed comparably and the local effective tumbling times for individual residues are plotted above. Shown here are the distributions obtained using data collected at 600 MHz (^1H) (micelles, **C**) and 800 MHz (^1H) (bicelles, **D**).

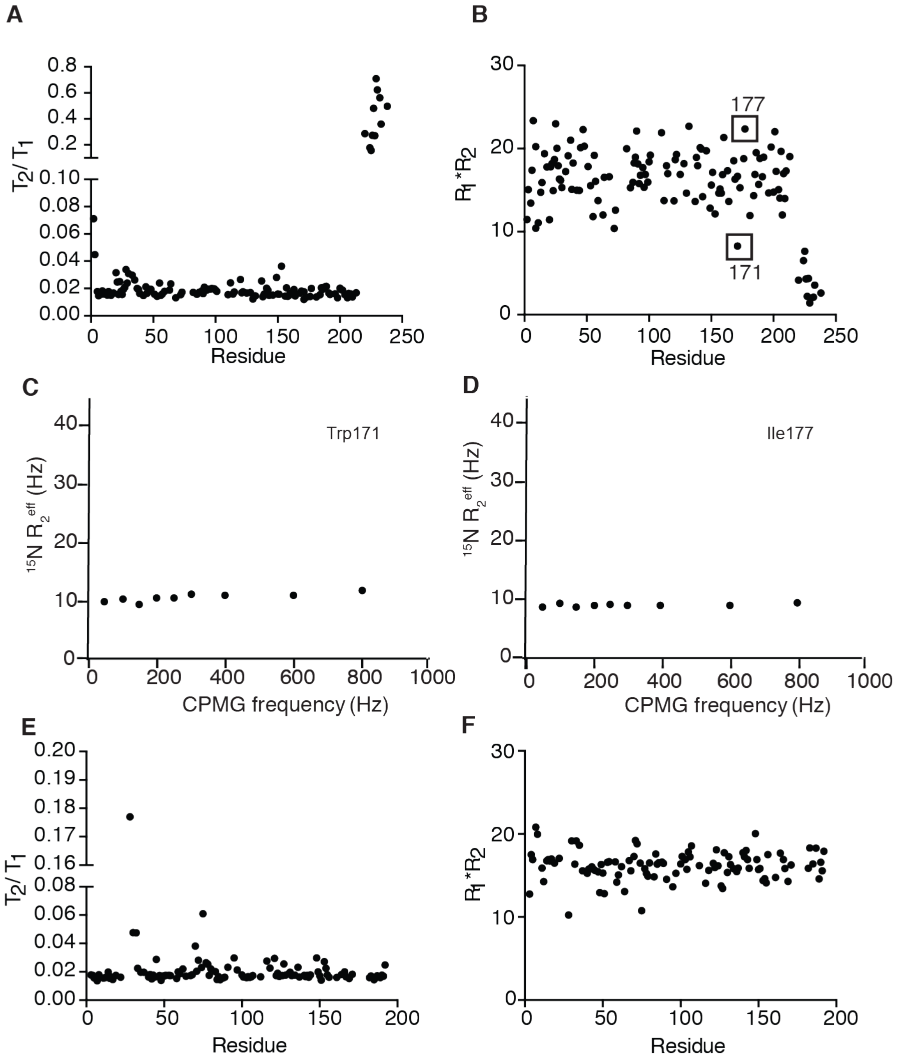

Fig. S4. The helical and sheet backbones of pSRII and OmpW are largely dynamically silent. (**A**) T_1/T_2 ratios and (**B**) R_1*R_2 products averages for experiments collected on pSRII at both 600 and 750 MHz. Example CPMG dispersion curves are shown in (**C**) and (**D**) for example outlier residues in the R_1*R_2 product (**B**) demonstrating a lack of intermediate-timescale exchange motions in pSRII. (**E**) T_1/T_2 ratios and (**F**) R_1*R_2 products for experiments collected on OmpW at 600 MHz demonstrate a comparable lack of fast and intermediate timescale motions.

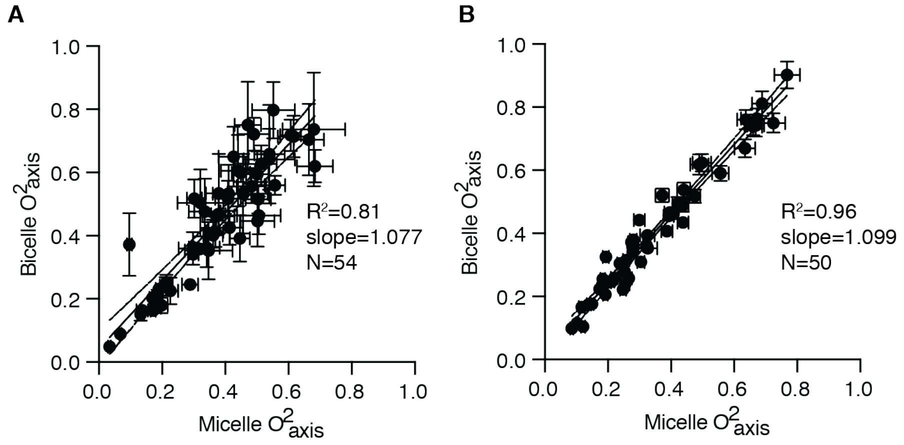

Fig. S5. Correlation of O^2_{axis} values for pSRII & OmpW in detergent micelles & lipid bilayer bicelles. Both pSRII (**A**) and OmpW (**B**) show strong linear correlations of experimentally determined methyl O^2_{axis} values when incorporated into detergent micelles and lipid bilayers. The O^2_{axis} value for all methyl groups that are confidently mapped in both sample conditions and that display reasonable error values (< ±0.20) are plotted with horizontal and vertical error bars determined by Monte Carlo sampling[34]. Linear fit statistics are shown for each. Errors are much higher in bicelle-incorporated pSRII due to incomplete levels of deuteration that results in competing dipolar relaxation with residual ^{1}H.

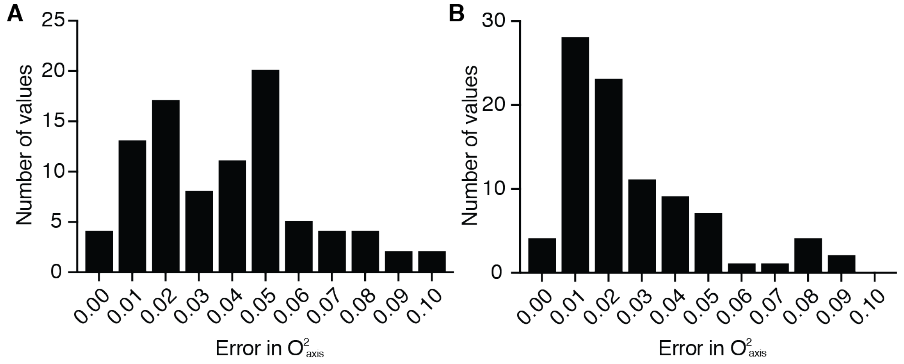

Fig. S6. Distribution in errors in O^2_{axis} values estimated by Monte Carlo sampling for pSRII at 50 °C (**A**) and OmpW at 40 °C (**B**) incorporating experimental fitting error and error in global tumbling time. All O^2_{axis} values used for this analysis have an error less than 0.10 and have an average error of 0.038 (pSRII) and 0.019 (OmpW).

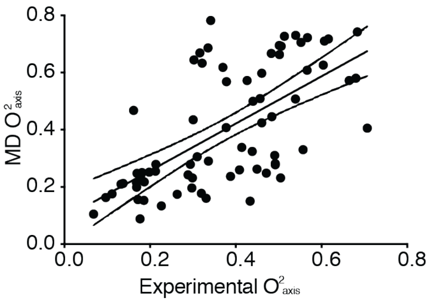

Fig. S7. Comparison with O^2_{axis} values derived from molecular dynamics simulations of pSRII. Linear correlation of molecular dynamics derived O^2_{axis} values with those obtained experimentally using cross-correlated relaxation NMR relaxation. The agreement is relatively poor (R^2 = 0.41, r.m.s.d = 0.16, slope = 0.83, y-intercept = 0.09) and is at the lower end of reliability of similar simulations for soluble proteins.[22b] The 95% confidence windows for the line of best fit are shown surrounding the main fit.

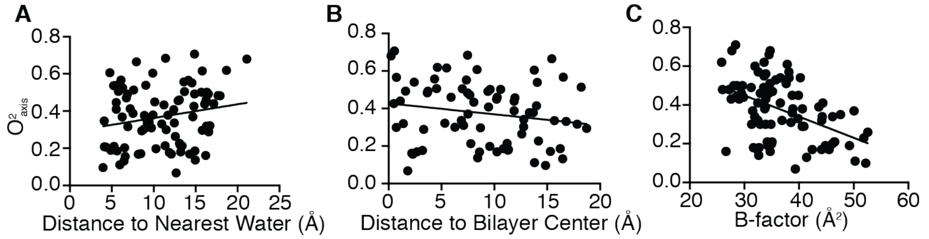

Fig. S8. Potential structural correlates to observed methyl dynamics in pSRII. (**A**) Correlation of methyl group motion with distance of the methyl carbon to the nearest water oxygen at the molecular surface (R^2 = 0.04). (**B**) Correlation of methyl group motion with distance of the methyl carbon to the putative bilayer center. (**C**) Correlation of methyl group motion with crystallographic B-factor (R^2 = 0.214). The crystal structure[18] (PDB code 1H68) was used. The analysis for depth of burial of each probe was carried out with the TravelDepth program[35]. Shown are the best-fitted lines to each data set.

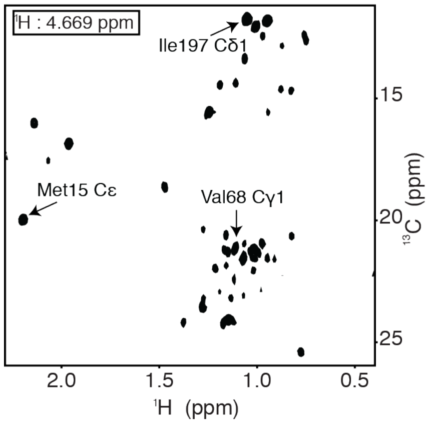

Fig. S9. Protein-water contacts. Two-dimensional plane at the water resonance of a three-dimensional methyl ^{13}C-resolved ^{1}H-^{1}H NOESY spectrum. Most peaks arise from solvent-exposed methyl groups, while the boxed residues of interest represent deeply buried methyl groups within close proximity to both structural water networks discussed in the text.

References

[1] E. S. O'Brien, D. W. Lin, B. Fuglestad, M. A. Stetz, T. Gosse, C. Tommos, A. J. Wand, *J. Biomol. NMR* **2018**, *72*, 263-273

[2] aA. Gautier, J. P. Kirkpatrick, D. Nietlispach, *Angew. Chem. Int Ed. Engl.* **2008**, *47*, 7297-7300; bA. Gautier, H. R. Mott, M. J. Bostock, J. P. Kirkpatrick, D. Nietlispach, *Nat. Struct. Mol. Biol.* **2010**, *17*, 768-774.

[3] N. K. Burgess, T. P. Dao, A. M. Stanley, K. G. Fleming, *J. Biol. Chem.* **2008**, *283*, 26748-26758.

[4] N. K. Goto, K. H. Gardner, G. A. Mueller, R. C. Willis, L. E. Kay, *J Biomol. NMR* **1999**, *13*, 369-374.

[5] C. P. Moon, N. R. Zaccai, P. J. Fleming, D. Gessmann, K. G. Fleming, *Proc. Natl. Acad. Sci. USA* **2013**, *110*, 4285-4290.

[6] K. H. Gardner, L. E. Kay, *J. Am. Chem. Soc.* **1997**, *119*, 7599-7600.

[7] J. DiFabio, S. Chodankar, S. Pjerov, J. Jakoncic, M. Lucas, C. Krywka, V. Graziano, L. Yang, in *Proceedings of the 12th International Conference on Synchrotron Radiation Instrumentation, Vol. 1741* (Eds.: Q. Shen, C. Nelson), **2016**.

[8] A. Gautier, D. Nietlispach, *Methods Mol. Biol.* **2012**, *914*, 25-45.

[9] S. G. Hyberts, A. G. Milbradt, A. B. Wagner, H. Arthanari, G. Wagner, *J. Biomol. NMR* **2012**, *52*, 315-327.

[10] F. Delaglio, S. Grzesiek, G. W. Vuister, G. Zhu, J. Pfeifer, A. Bax, *J. Biomol. NMR* **1995**, *6*, 277-293.

[11] N. A. Lakomek, J. Ying, A. Bax, *J. Biomol. NMR* **2012**, *53*, 209-221.

[12] J. Cavanagh, W. J. Fairbrother, A. G. Palmer, M. Rance, N. J. Skelton, *Protein NMR Spectroscopy: Principles and Practice*, Elsevier, **2007**.

[13] J. P. Loria, M. Rance, A. G. Palmer, 3rd, *J. Biomol. NMR* **1999**, *15*, 151-155.

[14] D. M. Korzhnev, K. Kloiber, V. Kanelis, V. Tugarinov, L. E. Kay, *J. Am. Chem. Soc.* **2004**, *126*, 3964-3973.

[15] H. Sun, L. E. Kay, V. Tugarinov, *J. Phys. Chem. B* **2011**, *115*, 14878-14884.

[16] K. A. Sharp, V. Kasinath, A. J. Wand, *Proteins* **2014**, *82*, 2106-2117.

[17] Y. Fu, V. Kasinath, V. R. Moorman, N. V. Nucci, V. J. Hilser, A. J. Wand, *J. Am. Chem. Soc.* **2012**, *134*, 8543-8550.

[18] A. Royant, P. Nollert, K. Edman, R. Neutze, E. M. Landau, E. Pebay-Peyroula, J. Navarro, *Proc. Natl Acad. Sci. USA* **2001**, *98*, 10131-10136.

[19] W. Humphrey, A. Dalke, K. Schulten, *J. Mol. Graph.* **1996**, *14*, 33-38, 27-38.

[20] J. C. Phillips, R. Braun, W. Wang, J. Gumbart, E. Tajkhorshid, E. Villa, C. Chipot, R. D. Skeel, L. Kale, K. Schulten, *J. Comput. Chem.* **2005**, *26*, 1781-1802.

[21] W. L. Jorgensen, J. Chandrasekhar, J. D. Madura, R. W. Impey, M. L. Klein, *J Chem Phys* **1983**, *79*, 926-935.

[22] aV. Kasinath, K. A. Sharp, A. J. Wand, *J. Am. Chem. Soc.* **2013**, *135*, 15092-15100; bE. S. O'Brien, A. J. Wand, K. A. Sharp, *Protein Sci.* **2016**, *25*, 1156-1160.

[23] R. Horst, P. Stanczak, K. Wuthrich, *Structure* **2014**, *22*, 1204-1209.

[24] J. Lee, X. Cheng, J. M. Swails, M. S. Yeom, P. K. Eastman, J. A. Lemkul, S. Wei, J. Buckner, J. C. Jeong, Y. F. Qi, S. Jo, V. S. Pande, D. A. Case, C. L. Brooks, A. D. MacKerell, J. B. Klauda, W. Im, *J. Chem. Theory Comp.* **2016**, *12*, 405-413.

[25] aJ. B. Klauda, R. M. Venable, J. A. Freites, J. W. O'Connor, D. J. Tobias, C. Mondragon-Ramirez, I. Vorobyov, A. D. MacKerell, Jr., R. W. Pastor, *J. Phys. Chem. B* **2010**, *114*, 7830-7843; bN. Kucerka, Y. Liu, N. Chu, H. I. Petrache, S. Tristram-Nagle, J. F. Nagle, *Biophys. J.* **2005**, *88*, 2626-2637.

[26] D. M. York, W. T. Yang, *J Chem Phys* **1996**, *104*, 159-172.

[27] A. L. Lee, S. A. Kinnear, A. J. Wand, *Nat. Struct. Biol.* **2000**, *7*, 72-77.

[28] A. L. Lee, A. J. Wand, *Nature* **2001**, *411*, 501-504.

[29] K. K. Frederick, M. S. Marlow, K. G. Valentine, A. J. Wand, *Nature* **2007**, *448*, 325-329.

[30] H. Okamura, K. Makino, Y. Nishimura, *J Mol Biol* **2007**, *367*, 1093-1117.

[31] V. Tugarinov, L. E. Kay, *Biochemistry* **2005**, *44*, 15970-15977.

[32] W. Liu, P. F. Flynn, E. J. Fuentes, J. K. Kranz, M. McCormick, A. J. Wand, *Biochemistry* **2001**, *40*, 14744-14753.

[33] V. R. Moorman, K. G. Valentine, A. J. Wand, *Protein Sci.* **2012**, *21*, 1066-1073.

[34] M. A. Stetz, J. A. Caro, S. Kotaru, X. J. Yao, B. S. Marques, K. G. Valentine, A. J. Wand, in *Biological Nmr, Pt B, Vol. 615* (Ed.: A. J. Wand), **2019**, pp. 237-284.

[35] R. G. Coleman, K. A. Sharp, *J Mol Biol* **2006**, *362*, 441-458.
